# Effects and mechanisms of polysaccharides from natural medicinal plants on improving aerobic exercise capacity

**DOI:** 10.3389/fnut.2025.1650499

**Published:** 2025-09-01

**Authors:** Mingxin Xu, Weiyu Chen, Wenjian Liang

**Affiliations:** ^1^Guangzhou University of Chinese Medicine, Guangzhou, China; ^2^The Fifth Clinical College of Guangzhou University of Chinese Medicine, Guangzhou, China; ^3^Guangdong Second Traditional Chinese Medicine Hospital, Guangzhou, China

**Keywords:** natural medicinal plant polysaccharides, aerobic exercise capacity, bioactivity mechanisms, structure-activity, anti-fatigue mechanisms

## Abstract

Aerobic exercise capacity is a critical determinant of endurance performance and overall health. Natural medicinal plant polysaccharides (NMPPs) have emerged as promising bioagents to enhance aerobic capacity through multi-target mechanisms. This review summarizes the effects of NMPPs on improving aerobic capacity, including oxygen supply and utilization in skeletal muscle, as well as the storage and metabolism of energy substrates. Additionally, we discuss the structural specificity related to their bioactivities. Furthermore, the mechanisms by which NMPPs enhance aerobic capacity encompass anti-fatigue properties, antioxidative effects, anti-inflammatory actions, immunomodulation, and modulation of gut microbiota. However, although there are many *in vitro* evidences, clinical translation requires standardized human trials and deeper exploration of structure-activity relationships. NMPPs represent a safe, multi-mechanistic alternative to conventional strategies, offering novel solutions for improving athletic performance and health resilience.

## 1 Introduction

Aerobic exercise capacity refers to the body's ability to efficiently produce energy via aerobic metabolism during sustained exercise to maintain muscle contractions. Its key physiological indicators are VO_2_ max and lactate threshold (1). In competitive sports, excellent aerobic exercise ability is closely related to the endurance of athletes (2). Highly trained male runners exhibited enhanced running economy relative to amateur-level runners (3). Aerobic exercise is an effective non-pharmacological intervention to improve cardiorespiratory fitness (CRF) (2). Enhanced aerobic capacity has been associated with diminished morbidity, decrease risk, and improved quality of life for cardiovascular diseases. Epidemiological studies indicate that long - term inactivity can cause a marked decrease in cardiopulmonary function (VO_2_ max). Around 28%−35% of adults have a VO_2_ max below the health threshold (< 35 ml/kg/min). The primary interventions currently employed to enhance aerobic exercise capacity involve specific training methodologies, including high-volume training (HVT), threshold training (THR), high-intensity interval training (HIIT), as well as nutritional supplements such as creatine. However, the practical implementation of these established training regimens often faces challenges related to poor adherence in the general population, primarily due to the substantial time commitment, perceived discomfort, or difficulty associated with maintaining high training volumes or intensities (4). Furthermore, conventional nutritional supplements may interfere with exercise adaptability and yield only limited improvements in CRF (5). Critically, even when adhered to, improvements in aerobic capacity achieved through these common interventions are frequently reported as modest and not consistently sustained (6). Therefore, exploring novel, safe, and bioactive strategies that can effectively augment the adaptive response to exercise training represents a crucial research imperative.

NMPPs are novel natural origin nutritional agents, and have the potential for multi-target physiological regulation to enhancing aerobic exercise capacity. NMPPs are biopolymers formed by the connection of more than 10 monosaccharides through glycosidic bonds and are widely found in the roots, stems, or fruits of medicinal plants such as *Astragalus membranaceus, Lycium barbarum*, and *Ganoderma lucidum*, etc. They are attracting increasing attention due to their diverse bioactivities, including antioxidative properties, anti-inflammatory effects, immune regulation, and so on. Their activity is closely related to the molecular properties, including monosaccharide composition, degree of branching, and spatial conformation (7). Recent studies have revealed that NMPPs exert anti-fatigue effects through pathways such as scavenging free radicals induced by exercise, regulating energy metabolism, and inhibiting inflammatory cascade reactions (8–10), which is closely related to aerobic exercise performance. Although current studies in this area are not as extensive as those examining other functions, the implications for improving athletic performance remain significant. This review aims to summarize the effects and mechanisms of NMPPs in improving aerobic exercise capacity, fully exploring the potential of polysaccharides to provide new solutions for enhancing aerobic exercise capacity.

## 2 Classification and biological activities of polysaccharides from natural medicinal plants

### 2.1 The classification of natural medicinal plant polysaccharides

NMPPs can be classified by different criteria, including resources, solubility, extraction methods, and structural characteristics. Among these, structural classification is one of the most significant determinants of bioactivity. The molecular structures encompass various factors, including molecular weight, monosaccharide compositions, structural characterization, types of modification of natural polysaccharides and conformational characterization (9). Among these structural features, functional groups significantly influence their bioactivities by interacting with biological receptors and regulating signaling pathways. This interaction occurs through alterations in the charge distribution, spatial conformation, and hydrophilicity/hydrophobicity of the polysaccharides. Here, NMPPs with five common functional groups are discussed.

#### 2.1.1 Sulfated polysaccharides

Sulfated polysaccharides (SPs) are naturally occurring anionic polymers primarily composed of cellulose and hemicellulose, characterized by sulfate ester groups (${\text{-OSO}}_{3}^{-}$) as a class of bioactive macromolecules in plant systems (11, 12). Latest studies have provided an update on the structural chemistry of the major sulfate polysaccharides, including the galactans (e.g., agarans and carrageenans), ulvans, and fucans. SPs have demonstrated numerous beneficial bioactivities, including antioxidant, antidiabetic, hypoglycemic, anti-inflammatory, immunomodulatory, antiviral, and anticancer effects (13). The sulfated *Morinda citrifolia* (14) and *Chinese yam* (15) polysaccharide showed the good antioxidant activity, and them up to Vc level. The SPs from *Orchis chusua D. Don* maintained moderately stable antioxidant and probiotic ability. Among the various bioactivities, the modulation of adaptive immunity by SPs through multiple mechanisms has been the most extensively studied. Polysaccharides from Sea buckthorn leaves (SBLPs) are sulfated polysaccharide containing uronic acid. SBLPs showed antioxidant activity and immunological activity *in vitro*, also had the activity of immune stimulation on RAW264.7 cell (16). SPs promote dendritic cell maturation and antigen presentation to initiate T cell responses, directly regulate T cell activation, proliferation, and differentiation while balancing T cell subsets (e.g., Th1, Th2, Th17, and Treg), stimulate B cell activation and antibody production, enhance NK cell cytotoxicity, and induce cytokine secretion (e.g., ILs, IFNs, and TNF-α) to coordinate immune responses. These combined mechanisms enhance pathogen/tumor clearance, suggesting SPs hold significant promise as adjuvants in vaccine formulations (17).

Chemical modification can improve the physicochemical and functional properties of SPs. *Platycodon grandiflorum roots* polysaccharides (PGPs) exhibited specific antioxidant activities through Sephacryl S-100 column elution (18). The sulfated derivative polysaccharide from *Siraitia grosvenorii* had the ability to scavenge DPPH radicals, hydroxyl radicals and superoxide anions, and the scavenging power tended to increase with the increase in polysaccharide concentration (19). Natural *Lycium barbarum* seed dreg polysaccharides by sulfation showed the highest ABTS radical scavenging and reducing power while showed better DPPH radical scavenging effect than natural polysaccharides (20). Sulfated *plumula nelumbinis* polysaccharide significantly increase the proliferation of RAW264.7 macrophages and improve the activity of superoxide dismutase (SOD) and glutathione peroxidase (GSH-Px) based on cell model of H_2_O_2_-induced oxidative damage (21). However, most studies on biological effects of SPs have been conducted *in vitro* or in animal models. Therefore, further research involving human subjects is imperative to confirm these effects.

#### 2.1.2 Acetylated polysaccharides

The acetylated polysaccharides are characterized by that the sugar unit hydroxyl is replaced by an acetyl group (-OCOCH_3_). In nature, acetylated polysaccharides are extensively distributed in plants, microorganism, and animals (22). Acetyls play an essential role in polysaccharide behaviors for various biological activities (23). In recent years, a large number of studies have shown that polysaccharides exhibit excellent antioxidant and immune activities in the presence of acetyl groups. The antioxidant activity of *Litchi* pericarp polysaccharide (24), *Chinese yam* polysaccharide (15), *Cyclocarya paliurus* leaves polysaccharides (25) and pumpkin polysaccharides (26) were improved after acetylation modification. Introducing acetyls into polysaccharides could significantly modify their physicochemical properties and change their biological activities, such as solubility and water-solubility. After acetylation, *bitter gourd* polysaccharides (27), *cyperus esculentus* polysaccharides (28) exhibited stronger antioxidant, anticoagulant, and immune activity. The acetylated polysaccharide from *Orchis chusua D. Don* displayed the best proliferation effects on Bifidobacterium adolescentis (29). Arabinose (Ara) and galactose (Gal) contents were changed, and the antioxidant activity of Cyclocarya paliurus polysaccharide (CPP0.1) was subsequently increased (30). Acetylated polysaccharides of *Cyclocarya paliurus* polysaccharide have immunomodulatory effects on murine macrophage RAW264.7 (31). At the molecular level, pectic polysaccharide from *Cucurbita moschata Duch* likely activates macrophages mechanistically through TLR4- and CR3-dependent signaling pathways, involving coordinated activation of both NF-κB and MAPKs cascades (32).

#### 2.1.3 Carboxymethylated polysaccharides

Carboxymethylated polysaccharides contain carboxymethyl (-OCH_2_COOH), which can significantly enhance water solubility of native polysaccharides. This modification enables structural diversity as well as providing additional bioactivities. Carboxymethylated polysaccharides were found to exhibit antioxidant activity, anti-tumor activity, immunomodulatory activity and antibacterial activity (33). Among them, antioxidant activity has been studied more extensively. The carboxymethylated polysaccharides extracted from *peony seed dreg* maintained moderately stable antioxidant ability (34). Carboxymethylated *Morinda citrifolia* polysaccharide (14) and carboxymethylated polysaccharide from *Chinese yam* (15) showed the good antioxidant activity, and them up to Vc level. Carboxymethylated polysaccharide from *Orchis chusua D. Don* (29), carboxymethylated *cushaw* polysaccharide (35) and carboxymethylated *cucumber* polysaccharide (36) exhibit better ability to scavenge superoxide anions and hydroxyl radicals. In addition, carboxymethylation modification of relevant high degree of substitution can enhance the dendritic cells maturation-inducing function of polysaccharide from the seeds of *Plantago asiatica L* (37).

Carboxymethylation could effectively increase the antioxidant activities of the polysaccharide. Carboxymethylated polysaccharide from *Garcinia mangostana rind* showed stronger activity compared to the other three chemical modification (38). Carboxymethylated polysaccharides from *blackcurrant fruits* (CRNPs) possessed stronger scavenging activities on radicals (hydroxyl and superoxide radicals) and better anti-lipid peroxidation activities, as well as better protection effects on erythrocyte hemolyses *in vitro* compared with polysaccharide extracted from blackcurrant fruits (RNP). The activities of CRNPs were significantly enhanced with the increase of the degree of substitution (DS) (203). One study showed that both of exopolysaccharide LEP-1b and its carboxymethylated derivative CLEP-1b from a *Lachnum sp* ameliorated physical fatigue and extended exhaustive swimming time. Moreover, CLEP-1b demonstrated dose-dependent enhancement of anti-fatigue effects, most notably at 200 mg/kg (39). This suggests that the carboxymethylated polysaccharides can be exploited as a potential healthcare compound to combat fatigue and to boost strength. Even though many studies showed that carboxymethylation could enhance the bioactivities, the mechanisms by which the carboxylate group contributes to these bioactivities remain unclear. Further exploration of the interaction between carboxymethyl polysaccharides and body molecules will facilitate the targeted production of functional polysaccharides.

#### 2.1.4 Phosphorylated polysaccharides

Phosphorylated polysaccharides contain phosphate groups covalently attached to their saccharide units through ester bonds (${\text{-O-PO}}_{3}^{2}-$), imparting unique charge and functional properties (40). Phosphorylation can reduce viscosity, improve the water solubility and biological activity of natural polysaccharides. Therefore, phosphorylated polysaccharides have attracted increasing attention owing to their antioxidant, antitumor, antiviral, immunomodulatory, and hepatoprotective effects (41). Phosphoric *Onion* polysaccharides (42), phosphorylated *Morinda citrifolia* polysaccharide (14) and phosphorylated polysaccharide from *Chinese yam* (15) have a good antioxidant activity, and the activity was similar to that of Vc positive control. Moreover, phosphorylated polysaccharide from *Orchis chusua D. Don* (SP-P) was demonstrated the highest scavenging ability on hydroxyl radical and growth-promoting activity on Lactobacillus Bulgaricus (29).

Due to their diverse bioactivities and structural modifications, phosphorylated polysaccharides are increasingly studied as targets of phosphorylation modification. The phosphorylation modification product of polysaccharide from purple sweet potato (PPSP) could significantly enhances the scavenging effects on hydroxyl radicals and superoxide anions. Additionally, it could also improve the anti-lipid peroxidation ability (43). The phosphorylated polysaccharides from *peony seed dreg* exhibited maximum hydroxyl radical scavenging activity and ferrous ion chelating ability as compared to native polysaccharides (34). Phosphorylated *Cyclocarya paliurus* polysaccharide (P-CP) significantly boosted its ability to protect cells from hydrogen peroxide-induced oxidative damage compared to the native polysaccharide (44). Phosphorylation of polysaccharide from *Sanchi (Panax notoginseng) flower* (45), phosphorylation modification effectively enhances *Abrus cantoniensis* Polysaccharides (ACP) (46), phosphorylated *pumpkin* polysaccharide (47), phosphorylated polysaccharides from *native ginseng* (48), phosphorylated *garlic* polysaccharide (49), phosphorylated *cushaw* polysaccharides (50), the antioxidant activity of phosphorylated polysaccharides is several times stronger than the unphosphorylated polysaccharide. The phosphorylated derivatives of Amana edulis polysaccharide possess higher reducing power compared with the native compound (51). Phosphorylated *Radix Cyathulae officinalis Kuan* polysaccharides (PRCPs) have been extensively studied in immunoregulatory activity. PRCPs not only enhance humoral immunity by elevating serum immunoglobulin levels (IgG, IgA, IgM) and promoting splenocyte proliferation, but also strengthen cellular immunity through macrophage phagocytosis activation, cytokine modulation (IFN-γ, IL-2,−4,−5,−6,−10), and T-cell subpopulation regulation (52). Furthermore, PRCPs acts as a potent adjuvant to boost vaccine efficacy by facilitating dendritic cell maturation and amplifying pathogen-specific antibody responses (53, 54). The efficacy of a drug is closely related to its structure. Although phosphorylated polysaccharides are highly active and have a wide range of effects, the structure-activity relationship and mechanism of action have not been studied extensively.

#### 2.1.5 Amino polysaccharides

Amino polysaccharides contain amino groups, typically derived from amino sugar monomers. Chitosan is a typical and relatively extensively studied amino polysaccharide. Chitosan was widely used in food and pharmaceutical industry due to its multidimensional properties, such as biocompatibility, biodegradability, antibacterial properties and non-toxicity, muco-adhesivity, adsorption properties, etc., and thus they can be widely used in variety of areas (55–57). Chitosan and its nanocomposites have applications in drug delivery (58, 59) and carrier for fertilizer (60). Animal experiments have shown that the amino sugar/curdlan hybrid materials are promising as a new type of polysaccharide immunoadjuvants useful for cancer chemotherapy (61). The function of polysaccharides in the medical field and the mechanism of interaction with body molecules need to be further explored and studied.

It is postulated that bioactivities of polysaccharides and physicochemical properties are directly or indirectly regulated by their structure (62). By introducing groups into the polysaccharide chain through physical, biological and chemical molecular modifications, the functions and effects of polysaccharides can be better exerted (63).

### 2.2 The biological activity of polysaccharides from natural medicinal plants

#### 2.2.1 Anti-fatigue activity

The anti-fatigue activity of NMPPs is a multifaceted process involving direct enhancement of energy metabolism, reduction of fatigue-associated biomarkers, and activation of antioxidant defense systems, often through synergistic or multi-target mechanisms. Polysaccharides from *Zingiber officinale* (ZOPA) significantly improve glycogen storage in gastrocnemius muscles while regulating energy metabolism and reducing metabolic waste accumulation, thereby delaying fatigue onset (64). *Dendrobium officinale* polysaccharide (EPDO) extends forced swimming time in mice by downregulating blood lactic acid (BLA) and urea nitrogen (BUN) levels, coupled with elevated SOD activity, highlighting its dual role in metabolic regulation and oxidative stress mitigation (65). These effects are further exemplified by *okra* polysaccharides, which prolong swimming endurance by increasing hepatic and muscle glycogen reserves while suppressing BUN and BLA accumulation (66).

The structural specificity of NMPPs significantly influences anti-fatigue potency. *Bupleurum chinense DC* polysaccharide BCP-2 showed superior fatigue-alleviating effects compared with BCP-1, attributable to distinct backbone compositions and branching patterns. Both contain oligogalacturonides, but BCP-1 features a backbone of 4-β-Galp and 4,6-β-Glcp with C4-branching, whereas BCP-2 consists of 3,5-α-Araf residues branched at C3 (67). Acidic polysaccharides from *Panax ginseng* (e.g., WGPA-A and WSGP-S3) exhibit superior activity compared with neutral counterparts, attributed to their higher sulfate content and molecular conformation (68, 69). Such structure-activity relationships suggest that targeted modifications of polysaccharide physicochemical properties could optimize their anti-fatigue potential.

NMPPs-mediated antioxidant activity can enhance exercise capacity. Polysaccharides from *Gynostemma pentaphyllum* (GPP) prolong exercise endurance in mice by scavenging excess ROS and preserving skeletal muscle glycogen, highlighting their potential to counteract exercise-induced oxidative stress (70). A*cerola* cold-water soluble polysaccharides (ACWS) exert anti-fatigue effects *in vivo*, likely via ROS neutralization and energy metabolism optimization (71). *Polygonatum sibiricum* polysaccharide (PSP) exhibits remarkable antioxidant and anti-aging properties by reducing ROS levels and increasing antioxidant enzyme activities in skeletal muscle tissue. However, direct studies on NMPPs in post-exercise recovery remain limited, direct evidence linking polysaccharide-mediated antioxidant effects to post-exercise recovery remains sparse.

Emerging evidence also implicates gut microbiota modulation and neuroendocrine regulation in polysaccharide-mediated fatigue resistance. *Astragalus* polysaccharides (APs) alleviates chronic fatigue syndrome by restoring gut microbial homeostasis and metabolite profiles, while *Phragmites rhizoma* polysaccharide suppresses hypothalamus-pituitary-adrenal axis hyperactivation to mitigate stress-induced fatigue (72, 73). These findings collectively highlight the diverse mechanisms through which plant polysaccharides combat fatigue, ranging from molecular-level metabolic adjustments to systemic physiological adaptations, positioning them as promising candidates for improving aerobic exercise capacity and post-exercise recovery.

#### 2.2.2 Antioxidant activity

The antioxidant activity of NMPPs plays a pivotal role in mitigating oxidative stress through direct free radical scavenging, activation of endogenous antioxidant pathways, and modulation of gut microbiota. *Garlic* polysaccharide (GP), an inulin-type fructan, exhibits potent direct antioxidant activity by neutralizing reactive oxygen species (ROS) (74). *Artemisia ordosica* polysaccharide (AOP) demonstrates dose-dependent free radical scavenging capacity *in vitro* and enhances systemic antioxidant defenses *in vivo* by upregulating GSH-Px and SOD activities in rats (75).

Indirect antioxidant mechanisms often involve immunomodulation and gut microbiota regulation. APs enhance antioxidant responses in coral trout by improving intestinal morphology and modulating microbial communities, which synergistically reduce oxidative stress (74, 76). *Mulberry leaf* polysaccharide reverses cyclophosphamide-induced intestinal damage in chicks by restoring gut microbiota balance and enhancing immune-antioxidant crosstalk (77). Xylo-oligosaccharides combined with γ-irradiated APs amplify antioxidant capacity in broilers through microbiota-driven immunometabolic adaptations (78).

Notably, NMPPs frequently exhibit synergistic antioxidant effects. *Date seed* polysaccharide-derived selenium nanoparticles (MPS-NPs) display dual antioxidant and antibacterial properties, suggesting ROS scavenging and pathogen inhibition jointly alleviate oxidative damage (79). Additionally, LBP improves sub-health conditions in mice by simultaneously boosting antioxidant enzymes, immune function, and anti-fatigue activity, indicating interconnected pathways (80). Antioxidant capacity is a critical contributor to anti-fatigue efficacy, particularly given the excessive free radical generation during prolonged exercise. *Lycium barbarum* polysaccharide (LBP) and *Panax ginseng* acidic polysaccharide (WGPA) exhibit potent antioxidant effects, enhancing SOD activity and alleviating oxidative damage (81, 82). Notably, *Polygonatum kingianum* polysaccharides (PKPs) ameliorate fatigue by activating the NRF2/HO-1/NQO1 pathway, which synergistically enhances antioxidant defenses and mitochondrial biogenesis via AMPK/PGC-1α/TFAM signaling (83). This dual modulation of redox balance and energy metabolism underscores the potential of polysaccharides to accelerate post-exercise recovery by counteracting exercise-induced oxidative stress.

#### 2.2.3 Anti-inflammatory effects

The anti-inflammatory properties of NMPPs have been extensively investigated, demonstrating therapeutic potential in diverse inflammatory diseases through modulation of inflammatory signaling pathways and immune cell functions. In hepatic fibrosis models, GLP further inhibits hepatic stellate cell activation and extracellular matrix deposition by targeting TGF-β/Smad signaling, while regulating inflammation-, apoptosis-, and cell cycle-related proteins (84). APs mitigate lipopolysaccharide (LPS)-induced systemic inflammation by blocking NF-κB/MAPK signaling (85).

Notably, the anti-inflammatory effects of polysaccharides are closely linked to their regulation of the gut microenvironment. *Rattan Pepper* polysaccharide alleviates dextran sulfate sodium (DSS)-induced intestinal inflammation and depressive behavior via bidirectional modulation of the microbiota-gut-brain axis, mediated by gut microbiota remodeling and reduced inflammatory mediators (e.g., IL-1β, IL-6) (86). Likewise, *Abelmoschus manihot* polysaccharide enhances intestinal mucus barrier integrity by promoting the abundance of Akkermansia muciniphila, thereby attenuating intestinal inflammation (87). Additionally, *Ephedrae Herba* polysaccharide suppresses ovalbumin (OVA)-induced asthmatic airway inflammation by restoring the Th1/Th2 and Th17/Treg immune balance (88).

At the molecular level, polysaccharides predominantly exert anti-inflammatory effects by inhibiting key pathways such as NF-κB and TLR4. RG-I pectin-like polysaccharide from *Rosa chinensis* alleviates non-alcoholic steatohepatitis-related inflammation and fibrosis by disrupting HMGB1/TLR4/NF-κB signaling (89). Colon-targeted modified *ginseng* polysaccharides significantly reduce pro-inflammatory cytokines (TNF-α, IL-6, IL-1β) and suppress NF-κBp65/TRAF6 signaling (90). *Hippophae rhamnoides* polysaccharides (HRP) further protect intestinal barrier function by upregulating tight junction proteins (occludin, claudin-1) and mitigating inflammatory mediators (91). NF-κB, as a transcription factor, plays an important role in the regulation of proinflammatory cytokines such as TNF-α, IL-1, IL-6, and IL-8 (92). Moreover, Moreover, immune adaptations was related to physical improvement (93).

#### 2.2.4 Immunomodulation

NMPPs mediate diverse immunomodulatory activities via direct cellular interactions, indirect signaling cascades, and synergistic effects. Direct immunomodulation is often mediated by enhancing immune cell activity or cytokine production. *Sophora cassia* polysaccharides significantly enhance B-cell and T-cell lymphocyte proliferation, indicating their potential to strengthen physical immunity (94). *Hippophae rhamnoides* polysaccharide (HRP) and *Apocynum venetum* flower polysaccharide (AVFP) demonstrate strong immune-enhancing effects *in vitro* and *in vivo*, likely by activating macrophages and lymphocytes (91, 95). A fructan-type GP further upregulates immune responses in macrophages and immunosuppressed mice, suggesting its role in restoring immune homeostasis (96). Additionally, polysaccharides from *Areca catechu L. inflorescence* effectively modulate immune responses in peripheral blood and spleen, emphasizing their systemic immunoregulatory capacity (97).

Indirect immunomodulation frequently involves interactions with the gut microbiota and intestinal immunity. *Dendrobium officinale* polysaccharide (DOP) and APs regulate macrophage and lymphocyte functions while improving intestinal barrier integrity and microbiota composition, thereby amplifying immune defenses (76, 98). *Yupingfeng* polysaccharides enhance intestinal health in Macrobrachium rosenbergii by fortifying immunity, barrier function, and microbial balance in low-fishmeal diets (99). *Floccularia luteovirens* polysaccharides activate the immune system in immunosuppressed mice by reshaping gut microbiota and fecal metabolites (100).

Synergistic mechanisms combining antioxidant and immunomodulatory effects are also prominent. Mulberry leaf polysaccharide alleviates cyclophosphamide-induced intestinal damage and growth inhibition in chicks by simultaneously boosting antioxidant capacity, immune regulation, and microbiota modulation (77). APs enhances coral trout growth and immunity by improving antioxidant responses and intestinal microbiota (101). Notably, xylo-oligosaccharides and γ-irradiated APs synergistically enhance immune responses and antioxidant capacity in broilers, highlighting the interplay between redox balance and immune activation (78). Additionally, *Ficus carica* polysaccharide (FCPs) mitigate oxidative stress via ROS scavenging while enhancing hepatic glucose metabolism and dendritic cell-driven IL-6/IL-12 production, positioning it as a multifunctional phytochemical candidate for metabolic-immune axis modulation (102).

Furthermore, polysaccharides such as *Ephedra sinica* polysaccharide (ESP) and *Rehmannia glutinosa* polysaccharide (RGP) modulate mucosal immunity by reducing pro-inflammatory cytokines, protecting intestinal barriers, and balancing microbiota-immune crosstalk, offering potential strategies to mitigate exercise-induced gastrointestinal stress (103, 104). These findings underscore the multifaceted immunomodulatory roles of plant polysaccharides, positioning them as promising candidates for improving exercise resilience through immune and metabolic optimization.

#### 2.2.5 Modulation of gut microbiota

The modulation of gut microbiota represents a key mechanism by which natural medicinal plant polysaccharides influence host health, acting through both direct microbial interactions and indirect host-mediated pathways. *Zingiber officinale* derived polysaccharides (ZOPA and ZOPA-1) directly enhance intestinal flora diversity, alter microbial abundance, and regulate short chain fatty acid (SCFA) concentrations, potentially mediating anti-fatigue effects through the gut-muscle axis (64). Ethanol precipitated polysaccharides from *Dendrobium officinale* (EPDO-60) restores oxidative-antioxidative balance and accelerates fatigue metabolite clearance by reshaping gut microbial community structure (65). These direct modulatory effects are often coupled with indirect mechanisms. *Green radish* polysaccharides and vinegar-processed *Schisandra chinensis* polysaccharide ameliorate hyperlipidemia and type 2 diabetes by promoting SCFAs production and regulating microbial composition, thereby improving metabolic homeostasis (105, 106). SCFAs, as critical metabolites, not only enhance intestinal barrier function but also serve as energy substrates for skeletal muscles, suggesting a potential link to aerobic exercise recovery by mitigating exercise-induced energy depletion.

Structural specificity also dictates functional outcomes. Steaming duration alters *Polygonatum cyrtonema* polysaccharide (PCP) molecular weight and monosaccharide composition, thereby diversifying their digestion, absorption, and fermentation characteristics by gut microbiota (107). Xylo-oligosaccharides combined with γ-irradiated APs synergistically enhance antioxidant capacity and microbiota composition in broilers (78), emphasizing the importance of structural optimization for targeted efficacy.

Some polysaccharides exert synergistic effects through multi-target pathways. PKPs alleviate fatigue by simultaneously activating NRF2/HO-1/NQO1 and AMPK/PGC-1α/TFAM signaling pathways while modulating gut microbiota (83). Similarly, APs improve chronic fatigue syndrome by regulating gut microbiota and metabolites, highlighting a microbiota-metabolite-axis crosstalk (73). This dual regulation underscores the interconnected roles of microbial balance and host signaling in enhancing physiological resilience.

Antioxidant and anti-inflammatory activities further link gut microbiota modulation to aerobic exercise adaptation. MPS-NPs exhibit dose-dependent antioxidant and antibacterial effects (79), while acid-assisted *Asparagus cochinchinensis* polysaccharides protect against neurodegeneration via the microbiota-gut-brain axis (108). Since intense exercise generates excessive ROS, polysaccharides with ROS-scavenging properties, such as AOP (75), may accelerate post-exercise recovery by neutralizing oxidative stress.

Emerging evidence suggests cross-tissue communication mediated by gut microbiota. PCPY-1, a homogeneous polysaccharide from *Polygonatum cyrtonema*, alleviates fatigue in exhausted mice by enhancing osteocalcin-mediated bone-muscle crosstalk, thereby promoting muscle energy metabolism and ATP generation (109). This highlights the potential of polysaccharides to bridge gut microbial modulation with systemic energy regulation, a mechanism highly relevant to aerobic endurance. Nevertheless, translational studies are required to validate these findings in exercise models, particularly regarding fatigue mitigation and performance enhancement.

#### 2.2.6 Hypoglycemic and hypolipidemic effects

Natural medicinal plant polysaccharides counteract hyperglycemia and hyperlipidemia by targeting metabolic pathways directly and leveraging gut microbiota-dependent mechanisms, with synergistic actions increasingly recognized. Direct regulation of glucose and lipid metabolism is often mediated by targeting key signaling pathways. *Rhizoma Ligustici Chuanxiong* polysaccharides (RLMP) enhance hepatic glucose uptake and suppress oxidative stress by activating the PI3K/Akt/GLUT-4 pathway, thereby reducing hyperglycemia in diabetic models (110). PSP improve insulin sensitivity, reduce glycated serum protein levels, and normalize lipid metabolism in T2DM mice, directly alleviating hyperglycemia and dyslipidemia (111). *Astragalus membranaceus* polysaccharides (AMP) further demonstrate direct anti-diabetic effects by restoring pancreatic β-cell function and inhibiting hepatic gluconeogenesis (112).

Due to the limited intestinal absorption of high-molecular-weight polysaccharides, their systemic benefits frequently rely on gut microbiota-mediated metabolic reprogramming. *Ulva lactuca* polysaccharides mitigate aging-associated hyperglycemia in diabetic mice by reshaping gut microbiota composition and promoting the production of SCFAs, which enhance insulin signaling and suppress systemic inflammation (113). *Ficus carica* polysaccharides, obtained via ultrasound-assisted extraction, ameliorate oxidative stress and immunomodulatory imbalances, likely through microbiota-dependent pathways involving bacterial metabolite interactions (102). *Green radish* polysaccharides alleviate diet-induced hyperlipidemia by enriching beneficial gut bacteria (e.g., Lactobacillus) and stimulating SCFA production, which suppresses hepatic lipid accumulation (106). These studies underscore the pivotal role of gut microbiota in translating polysaccharide structures into metabolic benefits.

Notably, certain polysaccharides exhibit synergistic mechanisms by combining direct metabolic effects with microbiota-dependent regulation. *Okra* polysaccharides alleviate T2DM by directly activating the PI3K/AKT/GSK3β-Nrf2 pathway to reduce oxidative stress while simultaneously modulating gut microbial ecology to improve glucose homeostasis (114). Such multi-target actions highlight the potential of polysaccharides to address complex metabolic disorders through complementary pathways. Although current research focuses on diabetes and hyperlipidemia, the antioxidant and anti-inflammatory properties of these polysaccharides may indirectly enhance aerobic exercise capacity. Exercise-induced oxidative stress and inflammation could be mitigated by polysaccharide-mediated free radical scavenging, potentially accelerating post-exercise recovery. However, more studies are needed to further explore the cross-mechanism interactions.

#### 2.2.7 Antitumor activity

Multifaceted antitumor properties of NMPPs arise from direct cytostatic/cytotoxic actions, indirect immune modulation and signaling pathway interference, and synergistic interplay often involving gut microbiota. A growing body of evidence highlights their ability to directly inhibit tumor cell proliferation and induce apoptosis. DOP suppress MNNG-induced precancerous lesions by regulating the Wnt/β-catenin pathway and altering endogenous metabolites (115). Similarly, APs inhibit tumor progression in prostate, liver, and non-small-cell lung cancers by suppressing cell growth, invasion, and enhancing apoptosis, while also improving chemosensitivity and immunity (116). *Crocus sativus* petal polysaccharides further demonstrate antitumor efficacy by remodeling the tumor microenvironment (TME) (117).

Indirect mechanisms often involve immune enhancement and gut microbiota modulation. *Glycyrrhiza* polysaccharides activate γδT cell-mediated antitumor responses via the TLRs/NF-κB pathway and gut microbiota interactions in murine models (118). A cold-water extracted polysaccharide-protein complex from *Grifola frondosa* exerts antitumor effects in H22 tumor-bearing mice by activating TLR4-NF-κB signaling and modifying gut microbiota composition (119). *Codonopsis* polysaccharides synergize with doxorubicin (DOX) to amplify tumor-killing effects and immune regulation (120).

Notably, gut microbiota-mediated pathways are a recurring theme. Reviews underscore that natural polysaccharides target gut microbiota to exert antitumor effects (121, 122). This aligns with broader evidence that polysaccharides modulate microbial metabolites, which in turn influence systemic immunity and inflammation—processes also critical in mitigating oxidative stress.

#### 2.2.8 Other activities

In addition to the well-documented bioactivities, natural medicinal plant polysaccharides exhibit diverse physiological effects, including anti-aging, and antidepressant activities, often mediated through gut microbiota modulation. Similarly, a water-soluble polysaccharide from *Ginkgo biloba leaves* exerts antidepressant activity by regulating the gut microbiome and its associated metabolites, suggesting a potential role in alleviating psychological stress through microbiota-gut-brain axis interactions (123).

These findings underscore the systemic and multifaceted roles of polysaccharides in promoting holistic health. While direct evidence linking these activities to aerobic exercise enhancement remains limited, their capacity to improve metabolic regulation, reduce oxidative damage, and modulate neuropsychiatric states may indirectly support exercise performance and recovery. Nevertheless, further studies are warranted to explore these potential cross-domain benefits and elucidate the mechanistic connections between polysaccharide modulation and aerobic capacity optimization.

**Figure 1:**
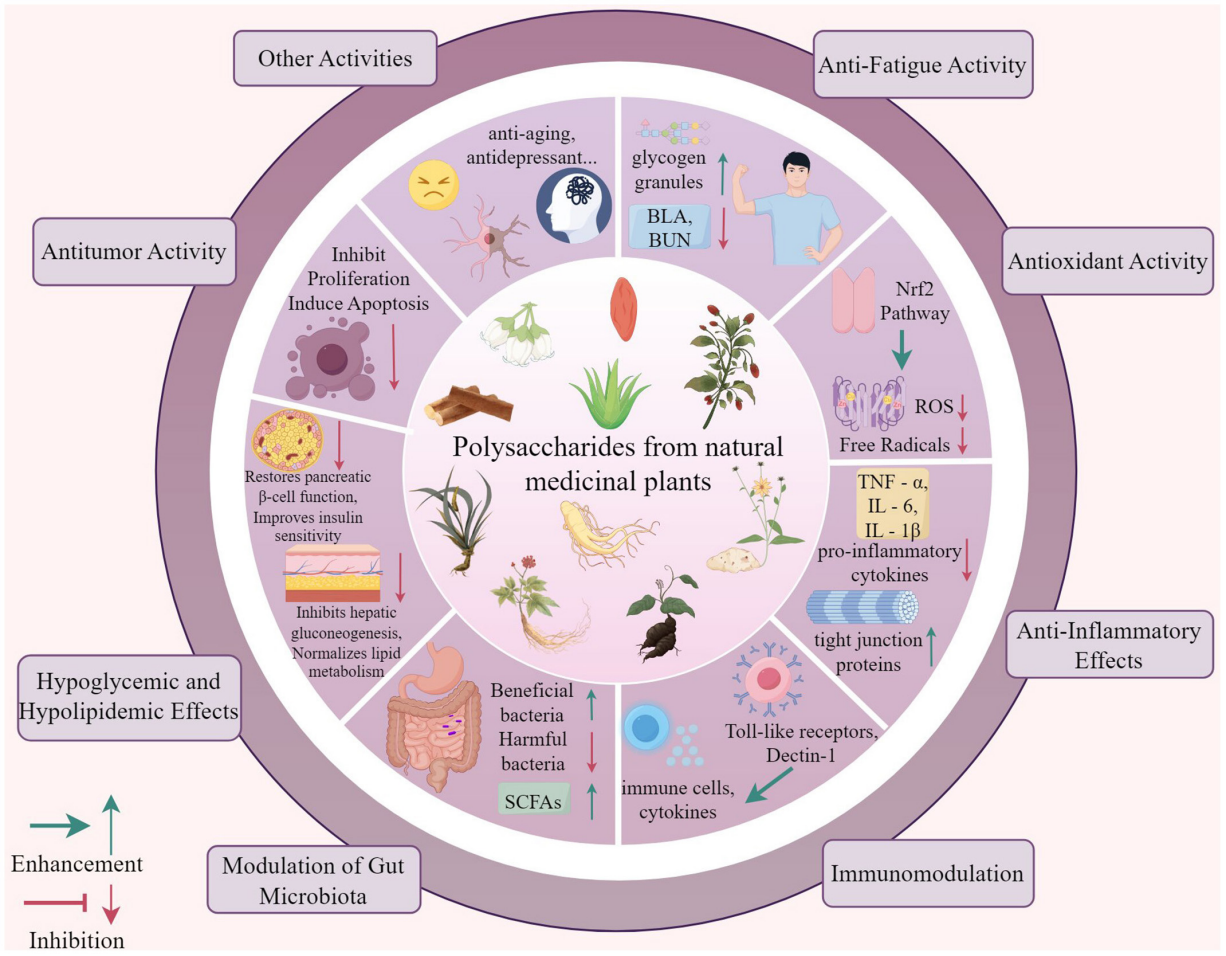
The biological activities of natural medicinal plant polysaccharides. The biological activity of polysaccharides from natural medicinal plants including anti-fatigue activity, antioxidant activity, anti-Inflammatory effects, immunomodulation, modulation of gut microbiota, hypoglycemic and hypolipidemic effects, antitumor activity, other activities like anti-aging and antidepressant activities.

NMPPs exhibit multifaceted bioactivities through multi-target and multi-pathway mechanisms, with their structural features dictating functional specificity. Through systematic induction and analysis, we have unveiled the extensive sources and diverse functions of these polysaccharides. Closely related to physiological foundations, these pathways enable medicinal plant polysaccharides to significantly ameliorate pathological states and enhance physiological conditions. These findings not only highlight their potential as supplements but also underscore their vast application value in modern medicine. Consequently, the role of medicinal plant polysaccharides as multifunctional natural supplements in the prevention and treatment of a variety of diseases cannot be overlooked, and their research and application prospects are promising. The biological activity of polysaccharides from natural medicinal plants is shown in Figure 1.

Table 1 provides a summary of the study on classification and biological activities of NMPPs.

**Table 1:** Study on classification and biological activities of natural medicinal plant polysaccharides.

**Functional group**	**Sources**	**Types**	**Biological activity**	**Author, Year**
**Sulfated Polysaccharides**	*Lycium barbarum seed dreg* polysaccharides	*In vitro*	**Antioxidant activity**	Xiu-Xiu Zhang et al., 2024, (20)
	*Platycodon grandiflorum* polysaccharides	*In vitro*		Wei Li et al., 2023, (18)
	Polysaccharides from *sea buckthorn leaves*	*In vitro*		Yang Liu et al., 2023, (16)
	Sulfated polysaccharide from *Siraitia grosvenorii*	Human hepatoma cells (HepG2), human breast cancer cells (MDA-MB-231), human non-small cell lung cancer cells (A549)		Pin Gong et al., 2023, (19)
	*Morinda citrifolia* polysaccharide	*In vitro*		Shiyang Zhou et al., 2022, (14)
	polysaccharide from Chinese yam	*In vitro*		Shiyang Zhou et al., 2021, (15)
	Polysaccharides from *Orchis chusua D. Don*	*In vitro*		Rehebati Nuerxiati et al., 2021, (29)
	Polysaccharides from *Plumula nelumbinis*	RAW264.7 macrophages		Yueping Jiang et al., 2018, (21)
	Polysaccharides from *sea buckthorn leaves*	RAW264.7 cell	**Immunomodulation**	Yang Liu et al., 2023, (16)
**Acetylated Polysaccharides**	*Litchi pericarp* polysaccharide	*In vitro*	**Antioxidant**	Yijie Wang et al., 2025, (24)
	Polysaccharides from *Cyperus esculentus*	RAW 264.7 cells		Huifang Wang et al., 2023, (28)
	*Morinda citrifolia* polysaccharide	*In vitro*		Shiyang Zhou et al., 2022, (14)
	Polysaccharide from *Chinese yam*	*In vitro*		Shiyang Zhou et al., 2021, (15)
	Polysaccharides from *Orchis chusua D. Don*	Bifidobacterium adolescentis		Rehebati Nuerxiati et al., 2021, (29)
	Acetylated *Cyclocarya paliurus* polysaccharide (Ac-CPP0.1)	*In vitro*		Meng-Zhao et al., 2021, (30)
	Polysaccharides from *Cyclocarya paliurus leaves*	*In vitro*		Jian-Hua Xie et al., 2015, (25)
	Polysaccharides isolated from pumpkin *(Cucurbita pepo, lady godiva)*	Rat thymic lymphocyte		Yi Song et al., 2013, (26)
	Polysaccharides from *Cyperus esculentus*	RAW 264.7 cell	**Immunomodulation**	Huifang Wang et al., 2023, (28)
	Pectic polysaccharide from *Cucurbita moschata Duch*	Macrophages		Linlin Huang et al., 2021, (32)
	Acetylated *Cyclocarya paliurus* polysaccharide	RAW 264.7 macrophages		Xin Liu et al., 2017, (31)
	Polysaccharides from *Cyperus esculentus*	RAW 264.7 cells	**Anti-inflammatory**	Huifang Wang et al., 2023, (28)
	Polysaccharides from *Smilax china L*	RAW 264.7 cells		Yu Zhang et al., 2019, (190)
	*Dendrobium officinale* polysaccharide	Mice with dextran sulfate sodium (DSS)-induced colitis	**Pro-prebiotic**	Yu Zhang et al., 2020, (191)
	Polysaccharides isolated from *pumpkin (Cucurbita pepo, lady godiva)*	Rat thymic lymphocyte	**Cytoprotective activity**	Yi Song et al., 2013, (26)
**Carboxymethylated Polysaccharides**	Polysaccharide from *Garcinia mangostana rind*	*In vitro*	**Antioxidant activity**	Zhenjie Tang et al., 2024, (38)
	*Morinda citrifolia* polysaccharide	*In vitro*		Shiyang Zhou et al., 2022, (14)
	Polysaccharide from *Chinese yam*	*In vitro*		Shiyang Zhou et al., 2021, (15)
	Polysaccharides from *Orchis chusua D. Don*	*In vitro*		Rehebati Nuerxiati et al., 2021, (29)
	Carboxymethylated *cushaw* polysaccharide	*In vitro*		L Yang et al., 2019, (35)
	Carboxymethylated and sulfated derivatives of *cucumber* polysaccharide	*In vitro*		S Chen et al., 2018, (36)
	Carboxymethylated polysaccharides of *peony seed dreg*	*In vitro*		Xiao-Li Li et al., 2009, (34)
	Polysaccharide from the seeds of *Plantago asiatica L*	Dendritic cells (DCs)	**Immunoregulatory activity**	Le-Ming Jiang et al., 2014, (37)
**Phosphorylated Polysaccharides**	Polysaccharide from *Sanchi (Panax notoginseng) flower*	*In vitro*	**Antioxidant activity**	Nailin Huo et al., 2022, (45)
	*Onion* polysaccharide	*In vitro*		Shiyang Zhou et al., 2022, (42)
	*Morinda citrifolia* polysaccharide	*In vitro*		Shiyang Zhou et al., 2022, (14)
	Polysaccharide from *purple sweet potato*	*In vitro*		Wenjian Yang et al., 2021, (43)
	Polysaccharide from *Chinese yam*	*In vitro*		Shiyang Zhou et al., 2021, (15)
	Polysaccharides from *Orchis chusua D. Don*	Lactobacillus Bulgaricus		Rehebati Nuerxiati et al., 2021, (29)
	Phosphorylated polysaccharide from *Cyclocarya paliurus*	RAW 264.7 cells		Liuming Xie et al., 2020, (44)
	Phosphorylated *pumpkin* polysaccharide	*In vitro*		Ling Chen et al., 2019, (47)
	Phosphorylated polysaccharide from *native ginseng*	*In vitro*		Xiong Xiong et al., 2019, (48)
	*Garlic* polysaccharide	*In vitro*		Junfan Chen et al., 2019, (49)
	Phosphorylated polysaccharides of *cushaw*	*In vitro*		Ling Chen et al., 2019, (50)
	Phosphorylated derivatives of *peony seed dreg* polysaccharide	*In vitro*		Xiao-Li Li et al., 2018, (34)

## 3 Aerobic exercise capacity and its influencing factors

The biological foundation of aerobic exercise capacity lies in the aerobic metabolic energy supply capacity. The direct energy source for skeletal muscle contraction during exercise is adenosine triphosphate (ATP), the resynthesis of which during this process primarily relies on aerobic metabolic pathways. The prerequisite for aerobic metabolic energy supply is oxygen availability. Consequently, any factor influencing oxygen supply or utilization within the organism can affect human aerobic metabolic energy production.

### 3.1 Oxygen supply capacity in skeletal muscle during exercise

The oxygen supply capacity of skeletal muscle during exercise depends on inspired oxygen concentration and cardiopulmonary function. VO_2_ max is an important variable that sets the upper limit for endurance performance and limited by the ability of the cardiorespiratory system to deliver oxygen to the exercising muscles (1). Increased vascular endothelial growth factor (VEGF) can induce angiogenesis, increase capillary density improve oxygen utilization, while elevated myoglobin further optimizes intracellular oxygen diffusion and improve blood supply to coronary arteries and skeletal muscle, then enhance the effect of endurance training (124). The increase of lactate and reactive oxygen species such as NO in the tissue can also cause vasodilation and increase the supply of blood and oxygen. Physiological levels of ROS are essential for skeletal muscle force production, while excess ROS induces contractile dysfunction. Muscle contraction activity increases the production of ROS through mitochondria, NADPH oxidase and other pathways (125).

### 3.2 Oxygen utilization capacity in skeletal muscle during exercise

During aerobic exercise, skeletal muscle utilizes oxygen, supporting energy substrate metabolism during contraction. Mitochondria generate ATP through the aerobic oxidation of carbohydrates, lipids and amino acids. Their oxidative capacity depends on mitochondrial density, cristae membrane surface area and the activity of aerobic enzymes (e.g. citrate synthase). Mitochondrial biogenesis is usually regulated by a variety of factors. PGC-1α is considered the main factor in regulating mitochondrial biogenesis, integrity and function, in cooperation with downstream nuclear transcription cofactors, such as nuclear respiratory factor-1 and−2 (NRF-1 and NRF-2) (126). PGC-1α expression is higher in tissues and organs with high energy metabolic load, such as heart, skeletal muscle and adipose tissue (127).

Mild heat shock plays an important role in metabolic remodeling by activating C2C12 muscle cell line mitochondrial biogenesis through the AMPK-SIRT1-PGC-1α axis (128). Notably, diverse exercise modalities similarly engage mitochondrial adaptation pathways, albeit with varying efficacy and molecular emphasis. Endurance training can enhance mitochondrial respiratory function in skeletal muscle by promoting the secretion of 12S rRNA-c (MOTS-c) and activating the AMPK/PGC-1α pathway (129). Endurance exercise for 8 weeks increased PGC-1α in the gastrocnemius muscle of rats, accompanied by increased mitochondrial biosynthesis and increased ratio of slow muscle fibers to fast muscle fibers (130). NOX2 and muscle/endothelial NOX4 can mediate skeletal muscle adaptation to endurance exercise through mitochondrial biogenesis and adaptive gene networks regulated by reactive oxygen species (131). HIIT can enhance the synthesis of mitochondrial proteins through DNA promoter region methylation, thereby improving mitochondrial respiration and aerobic capacity in skeletal muscle (132).

Antioxidant capacity plays an important role in maintaining mitochondrial function, which improves the body's ability to use oxygen and improve aerobic exercise capacity. Endurance exercise training increases the abundance of key antioxidant enzymes in the trained muscles, nuclear factor erythroid 2-related factor (Nrf2) signaling pathway is responsible for many of the exercise-induced changes in muscle antioxidant capacity (133).

### 3.3 Storage and metabolism of energy substances in skeletal muscle

Glucose and fat serve as two primary energy sources for prolonged exercise through aerobic metabolism, while the ability of skeletal muscles to oxidize glucose and fatty acids is also a crucial factor affecting aerobic endurance. Glycogen reserves in muscles and the liver can directly influence the magnitude of aerobic metabolic capacity and the duration of energy supply. The higher the glycogen content in muscles, the greater the potential for aerobic energy supply. Increased glycogen reserves in the liver help maintain stable blood glucose levels during prolonged exercise, thereby enhancing athletic performance. In addition, the ability of fat mobilization and liver utilization of glycerol, ketone bodies and some amino acids for gluconeogenesis are enhanced during long-term exercise, which also plays a very important role in improving long-term endurance exercise ability. Thus dietary intake during training influences the intensity and duration of exercise that athletes can sustain, ultimately affecting endurance performance. During exercise, supplementation with carbohydrates and other energy substrates can increase glycogen reserves in muscles. Strategic manipulation of carbohydrate and protein intake may optimize training adaptations, whereas excessive carbohydrate availability and antioxidants could blunt responsiveness (134). Plant polysaccharides have been shown to reduce fatigue and enhance athletic performance such as strength and endurance by improving energy metabolism (135, 136).

Emerging evidence suggests that fatty acids may regulate muscle lipid metabolism as signaling molecules through transcriptional mechanisms mediated by PPAR activation, NAD-dependent SIRT1 stimulation, and the AMPK signaling pathway (137). Exercise-induced cathecholamine release in skeletal muscle enhances the browning of white adipose tissue, promoting fat mobilization and improving aerobic metabolism (138). In addition, lactic acid produced by glycolysis may promote muscle metabolism toward lipid/glutamine oxidation through AMPK-PPAR activation, and enhance insulin sensitivity mediated by PI3K-AKT (139). Long-term intake of lactic acid and long-term high-intensity training can reduce body fat by increasing fat oxidation through lactic acid (140).

During exercise, exercise orchestrates a complex endocrine cascade that dynamically regulates energy substrate mobilization and utilization across tissues. The secretion of stress hormones such as adrenaline and catecholamine increases, and the release of muscle factors and cytokines from active muscles also play a hormone-like metabolic regulation role during long-term exercise, including stimulating glycogen breakdown in the liver and stimulating fat breakdown in adipose tissue. exercise-induced release of irisin from skeletal muscle enhances browning of white adipose tissue, indirectly supporting aerobic metabolism via systemic lipid mobilization (138). Exercise-induced activation of skeletal muscle p38γ stimulates interleukin-15 (IL-15) secretion, which signals to the motor cortex to enhance locomotor activity, forming a muscle-brain axis (141). IL-15, a skeletal muscle-derived myokine elevated during exercise, exhibits dual metabolic roles: pharmacological doses enhance systemic insulin sensitivity and lipid oxidation, while elevated interstitial muscle levels suggest autocrine/paracrine regulation of glucose homeostasis and oxidative metabolism, necessitating further investigation into dose-dependent signaling mechanisms (142). IL-6 increased GLUT4 expression in muscle and that this phenomenon may play a role in the post-exercise enhancement of insulin sensitivity in skeletal muscle (143).

## 4 Effects of polysaccharides from common natural medicinal plants on aerobic exercise capacity

### 4.1 *Ginseng* polysaccharides

*Ginseng* polysaccharides derived from the roots of Panax ginseng are well-studied for their medicinal properties including anti-fatigue effects. Studies have shown that the properties of polysaccharides affect their effects on improving aerobic exercise capacity. Water-soluble polysaccharides isolated from *Ginseng* (WGP) is an active component extracted from ginseng, which possesses a variety of pharmacological activities. WGPN (Neutral *Ginseng* Polysaccharide) and WGPA (Acidic *Ginseng* Polysaccharide) are two distinct fractions of WGP, respectively representing neutral and acidic polysaccharides. The anti-fatigue effects were evaluated using the forced swim test (FST), and serum biochemical parameters reduced immobility in the FST, but WGPA showed significant effects at lower doses compared to WGP and WGPN. Additionally, the FST-induced changes indicative of fatigue—such as decreased glucose (GLU) and GSH-Px levels, and increased creatine phosphokinase (CK), lactic dehydrogenase (LDH), and malondialdehyde (MDA) levels—were effectively mitigated by the respective doses of WGP, WGPN, and WGPA (69). It is worth noting that another study further fractionated WGPA into two components, WGPA-A and WGPA-N, using anion-exchange chromatography. In the forced swimming test, WGPA and WGPA-A were able to prolong the swimming time, whereas WGPA-N could not. Additionally, the levels of MDA and LDH in the serum were increased, while the levels of SOD and GSH-Px were decreased. Interestingly, the structural degeneration of mitochondria was ameliorated (81). WSGP-S3 is an acidic heteropolysaccharide extracted from steamed ginseng via ultrafiltration. In the anti-fatigue activity assays, WSGP-S3 significantly extended the exhaustive swimming time of fatigued mice. It also elevated the levels of liver and muscle glycogen, as well as the activities of SOD, catalase (CAT), and GSH-Px. Furthermore, it reduced the levels of BLA, BUN, and MDA compared to the control group. Additionally, WSGP-S3 promoted spleen cell proliferation in fatigued mice (68). The ginseng acidic polysaccharide APs-1 prolonged fatigue exhaustive swimming time, reduced BLA, LDH, and BUN levels, enhanced SOD and CAT activities, mitigated MDA-induced oxidative damage, increased CK activity, regulated glycolysis, and alleviated muscle fiber contraction (144). POL (Polysaccharide), OLI (Oligosaccharide), and WAT (Aqueous Extract) are different active components extracted from *Codonopsis pilosula*. Weight-loaded swimming test showed that, compared with the control treatment, only POL treatment significantly prolonged the swimming time of the mice. POL groups had the strongest hypoxia tolerance, followed by the OLI and WAT groups (145).

### 4.2 *Lycium barbarum* polysaccharide

LBP is one of the most extensively studied functional polysaccharides and has demonstrated various biological activities, including antioxidant, anti-fatigue, mitochondrial-enhancing, and energy metabolism-regulating properties. Recently, many studies have focused on its capacity to improve aerobic exercise performance through mechanistic insights into oxidative stress mitigation, mitochondrial function optimization, and innovative formulations for practical applications. In the exercise test, the rats treated with LBP showed a significantly prolonged time to exhaustion during running, along with a significant decrease in MDA levels and a significant increase in SOD and GSH-Px levels. These results indicate that LBP can effectively prevent oxidative damage following intense exercise (192).

In addition, LBP could enhance antioxidant ability in sub-health mice and showed anti-fatigue ability in sub-health mice (80). LBP-4a is a polysaccharide fraction purified from *Lycium barbarum*. After 4 weeks of treatment with LBP-4a in model mice prepared using compound factors such as forced swimming tests, sleep deprivation, and wrapping restraint stress tests, it was found that LBP-4a treatment reduced skeletal muscle damage and MDA levels, while enhancing SOD and GSH-Px activities compared to the model group. Additionally, LBP-4a increased mitochondrial membrane potential and calcium ion (Ca^2^^+^) levels in skeletal muscle mitochondria, with the high-dose group showing better effects than the low-dose group (146).

With the further research on LBP, it was gradually developed for practical application. LBP1-SeNPs are selenium nanoparticles (SeNPs) prepared using *Lycium barbarum* polysaccharide (LBP1) with a molecular weight of 92,441 Da as the stabilizer and capping agent. The high-dose group of LBP1-SeNPs exhibited the longest exhaustion swimming time, which was significantly greater than both the control group and the positive group. All tested dose groups of LBP1-SeNPs showed a significant increase in exhaustion swimming time compared to the control group, demonstrating that LBP1-SeNPs could be developed as a potential anti-fatigue nutritional supplement (147). *Lycium barbarum* polysaccharide effervescent tablets (LBPT) are effervescent tablets formulated by mixing LBP with excipients and help patients who have difficulty swallowing conventional tablets or capsules. Animal experiments showed that LBP and LBPT significantly increased the exhaustive swimming time in rats. LBP and LBPT improved biochemical markers in rat serum, such as lactic acid and creatine kinase, enhanced the antioxidant capacity of rat muscle, and reversed the decrease in serum glucose, ATP and glycogen content caused by exercise. Transmission electron microscopy showed that LBP and LBPT increased the density of mitochondria in rat liver (82). This suggests that more stable forms of polysaccharides can be mined and convenient dosage forms can be made for more people in the future.

### 4.3 Polygonatum sibiricum polysaccharide

*Polygonatum sibiricum* polysaccharide (PSP), derived from the rhizome of *Polygonatum sibiricum Redouté*, is a bioactive heteropolysaccharide distinguished by its unique monosaccharide composition (e.g., fructose, arabinose) and structural features. PSP effectively alleviated oxidative stress and mitochondrial dysfunction caused by D-gal in C2C12 myotubes, preserving mitochondrial integrity and reducing MAM formation. Additionally, PSP lowered intracellular Ca^2^^+^ levels by modulating calcium-related proteins, as confirmed by GO analysis of DEGs. In aged mice, PSP increased muscle mass, enhanced grip strength and hanging time, and reduced ROS levels while boosting antioxidant enzyme activities in skeletal muscle tissue (148).

*Polygonatum cyrtonema Hua* polysaccharide (PCP), another bioactive heteropolysaccharide extracted from the rhizome of *Polygonatum cyrtonema Hua*, is characterized by its complex structure composed of glucose, mannose, galactose, and highly branched configurations. PSP and PCP are not identical but distinct bioactive compounds derived from the *Polygonatum genus*. While both belong to the Liliaceae family and share structural similarities (e.g., glucose-rich heteropolysaccharides), their monosaccharide composition, glycosidic linkages, and bioactivity profiles differ due to species-specific genetic and metabolic variations. Pharmacological studies indicate PCP exhibits stronger mitochondrial function modulation and AMPK pathway activation compared to PSP, reflecting divergent mechanisms in enhancing aerobic capacity. In weight-loaded swimming test, PCP remarkably prolonged the exhaustive swimming time of mice, decreased serum levels of lactic acid (LA), blood urea nitrogen (BUN), SOD, GSH-Px and MDA, and increased the contents of liver glycogen, muscle glycogen and muscle ATP (149). It is worth noting that the swimming time and rotarod time in the high-dose group of PCP were significantly prolonged, increasing by 73 and 64%, respectively. The activities of CAT, GSH-Px and SOD in serum increased by 53.56, 37.69, and 53.67%, respectively, while the levels of MDA, lactic acid, and BUN decreased by 22.90, 17.48, and 24.61%, respectively (150). In the exhaustive swimming mouse model and the co-culture system of BMSCs/C2C12 cells, homogeneous polysaccharide (PCPY-1) from *Polgonatum cyrtonema* after structure characterization significantly stimulated BMSC differentiation into osteoblasts as determined by the protein expressions of osteogenic markers BMP-2, phosphor-Smad1, RUNX2, and osteocalcin. Meanwhile, PCPY-1 remarkably enhanced myoblast energy metabolism by upregulating osteocalcin release and GPRC6A protein expression; the phosphorylation levels of CREB and HSL; the mRNA levels of GLUT4, CD36, FATP1, and CPT1B, and ATP production *in vitro* and *in vivo* (109).

### 4.4 Maca (Lepidium meyenii Walp) polysaccharide

*Maca (Lepidium meyenii Walp.)*, a traditional medicinal plant native to the Andes, is rich in bioactive polysaccharides characterized by heterogeneous structural features, including distinct monosaccharide compositions and glycosidic linkages. Studies have demonstrated the anti-fatigue effects of Maca polysaccharides using the exhaustive swimming test and biochemical indexes (151), more in-depth research has further researched that the best dose of polysaccharide to exert effect. One study showed low-dose maca polysaccharides group (150 mg/kg/day) had the significant anti-fatigue activity (152). Another study revealed that mice treated with high-dose MP (100 mg/kg bw/day) exhibited significantly elongated swimming durations and accelerated average swimming speeds, along with improved serous biochemical parameters (153). This suggests that more in-depth studies could focus on the range between 100 and 150 mg/kg/day to explore the optimal dose of Maca polysaccharide to exert anti-fatigue.

Structural distinctions critical for polysaccharide function. Two fractions of polysaccharides, MPs-1 and MPs-2, were extracted from *Lepidium meyenii Walp.(maca)* using water and purified with DEAE-52 and Sephadex G-100 columns. MPs-2 (6.7 kDa) diverges from MPs-1 (7.6 kDa) in monosaccharide composition lacking xylose with a glucose-dominated ratio (1:1.3:36.8 vs. 1:1.7:3.3:30.5) and glycosidic linkage heterogeneity (mixed α/β-pyranose vs. exclusively α-configured). Both MPs-1 and MPs-2 have dose-dependent positive effects on fatigue-related parameters, with MPs-2 showing a better anti-fatigue effect than MPs-1 (154).

### 4.5 *Astragalus* polysaccharides

APs, primarily extracted from the roots of *Astragalus membranaceus* or *Astragalus mongholicus*, are heteropolysaccharides with diverse monosaccharide compositions (e.g., glucose, galactose, arabinose) and highly branched structures. APs plays a positive regulatory role in the proliferation and differentiation of sheep skeletal muscle satellite cells (SMSCs) (155). An optimal APs dose promotes growth, enhances antioxidant activity, supports immune function, and improves intestinal microbiota in coral trout (101). Moreover, there have been studies on the efficacy and mechanism of APs for Chronic fatigue syndrome (CFS) from the perspective of the gut-brain axis, APs could increase the SCFAs content by regulating the gut microbiota, and SCFAs (especially butyrate) can further regulate the oxidative stress and inflammation in the brain, thus alleviating CFS. This study provides a reference to further explore the efficacy of APs and the role of SCFAs in the central nervous system (73).

### 4.6 Other polysaccharides

Emerging evidence highlights that polysaccharides from diverse medicinal plants enhance aerobic exercise capacity by modulating energy metabolism, augmenting antioxidant defenses, and reducing fatigue-related biomarkers. *Dioscorea opposita* polysaccharides (PYB-1, PYB-2) prolong swimming endurance in mice via increased hepatic glycogen, elevated superoxide dismutase (SOD) and GSH-Px activities, and reduced malondialdehyde (MDA) levels (156). Similarly, *Zingiber officinale* (ZOPA, ZOPA-1), *Paris polyphylla* (PPPm-1), and *Gynostemma pentaphyllum* (GPP1-a) polysaccharides enhance skeletal muscle energy metabolism, improve contraction dynamics, and mitigate lactic acid accumulation (64, 70, 157). Polysaccharides from *Dendrobium officinale* (EPDO-60), *Bupleurum chinense* (BCP-2), *Apple pomace* (PAP), and *Cassiae semen* reduce BUN, LDH and oxidative stress while boosting glycogen reserves (65, 67, 94, 158). *Okra* (AEP-1, AEP-2), *Corn silk*, and *Mentha haplocalyx* (MHa) polysaccharides enhance ATPase activities (Na^+^-K^+^-ATP, Ca^2^^+^-ATP) and glycogen retention (66, 159, 160), while *Portulaca oleracea* alleviate exercise-induced fatigue markers (BUN, CK, MDA) and improve anti-fatigue effects (161). Structural features (e.g., molecular weight, purity) critically influence efficacy, as exemplified by PYB-1 had stronger free-radical scavenging activity than PYB-2 (156). These findings underscore the potential of natural polysaccharides as multi-target agents for enhancing exercise endurance through synergistic metabolic and antioxidative pathways.

Despite promising findings, several critical gaps persist in understanding the therapeutic potential of natural medicinal plant polysaccharides for enhancing aerobic exercise capacity. Current studies predominantly rely on animal models and acute exercise protocols, limiting translational relevance to chronic exercise adaptations and human physiology. Secondly, in terms of structure-activity relationships (SARs), exploring more specific structural features (such as linkage patterns, degree of branching, and molecular weight heterogeneity) could help enhance the effects of different polysaccharides. Furthermore, exploring a more precise dose-response relationship and stipulating a standardized protocol would be helpful for the practical application of polysaccharides. Additionally, long-term safety profiles, pharmacokinetic behavior, and bioavailability of polysaccharides in exercise contexts are poorly understood, hindering clinical translation. Future research should couple with advanced structural characterization techniques (e.g., NMR, AFM) to refine SAR models. Human trials are essential to validate preclinical findings, while formulation innovations, such as nanoencapsulation or synergistic combinatorial therapies, could enhance stability, targeting, and efficacy. Addressing these gaps will bridge mechanistic knowledge and practical applications, advancing polysaccharide-based strategies for optimizing aerobic performance.

The polysaccharides from natural medical plants on aerobic exercise capacity is shown in Table 2.

**Table 2:** Polysaccharides from natural medicinal plants on aerobic exercise capacity.

**Name**	**Source**	**Types**	**Testing Subjects**	**Number**	**Dose**	**Duration**	**Effects**	**Characteristics**	**References**
**PCP**	***Polygonatum cyrtonema Hua***	***In vivo***	**Seven-week-old male C57BL/6 mice (20 ± 2 g)**	**20**	**65, 130, 260 mg/kg/day**	**4 weeks**	**Body weight↑; Exhaustive swimming time↑**	**LA, BUN, SOD, GSH-Px, MDA↓; Liver glycogen, muscle glycogen, muscle ATP↑; BMP-2, phosphor-Smad1, Runx2, OC↑**	**(149)**
PCP	*Polgonatum cyrtonema Hua*	*In vivo*	Specific pathogen-free (SPF) male Kunming mice, aged 8 weeks and weighing 40 ± 2 g	15	65, 260 mg/kg/day	21 consecutive days	Exhausted swimming time↑	Serum activities of CAT, GSH-Px, SOD↑; MDA, lactic acid, BUN↓	(150)
PCPY-1	*Polygonatum cyrtonema Hua*	*In vivo*	Male C57BL/6 mice (7 months old; 30 ± 2 g)	10	65, 260 mg/kg/day	4 weeks	Exhausted swimming time↑	Osteocalcin release, GPRC6A protein expression↑; The phosphorylation levels of CREB and HSL↑; The mRNA levels of GLUT4, CD36, FATP1, CPT1B↑; ATP production↑	(109)
APs-1	*Panax ginseng C. A. Meyer*	*In vivo*	Male C57BL/6J mice (18–22 g)	8	50, 100, 150 mg/kg/day	15 days	Fatigue tolerance time↑	BLA, LDH, BUN↓; SOD, CAT↑, MDA↓, CK↑; LKB1, p-AMPK, PGC-1α, Glut4↑	(144)
WGP, WGPN, WGPA	*Panax ginseng C. A. Meyer*	*In vivo*	Male ICR mice, 11–12 weeks old	8	WGP:50, 100, 200 mg/kg; WGPA:40, 100, 160, 200 mg/kg; WGPN:40, 100, 160, 200 mg/kg	15 days	immobility in FST↓	GLU, GSH-Px↓; CK, LDH, MDA↑	(69)
WGPA, WGPA-A	*Panax ginseng C. A. Meyer*	*In vivo*	Male ICR mice, 11–12 weeks old	8	Dissolved in saline at a dose of 200 mg/kg and administered by oral gavage in a volume of 10 ml/kg	15 days	Forced swimming time↑	Malondialdehyde, lactate dehydrogenase↑; Superoxide dismutase, glutathione peroxidase↓; Ameliorate the structural degeneration of mitochondria	(81)
WSGP-S3	Steamed *ginseng*	*In vivo*	Male Kunming mice (20 ± 2 g)	10	25, 50, 75 mg/kg	30 days	Exhaustive swimming time↑	Liver and muscle glycogen levels, superoxide dismutase, catalase, glutathione peroxidase activities↑; BLA, nitrogen and malondialdehyde↓; Spleen cell proliferation↑	(68)
POL	*Codonopsis pilosula*	*In vivo*	Male ICR mice (SPF grade, 22 ± 2 g, 4 weeks old)	8	0.25, 0.5, 1.0 g/kg	21 consecutive days	Swimming time↑	LG, MG↑; BUN↓, LDH↓; MDA↓; GSH↑	(145)
LBP	*Lycium barbarum*	*In vivo*	Eight-week-old male Sprague-Dawley rats, weighing 280 to 300 g	12	100, 200 and 400 mg/kg	28 days	Mean endurance time of treadmill running to exhaustion↑	MDA↓; SOD, GSH-Px↑	(192)
LBP	*Lycium barbarum*	*In vivo*	Forty male Kun-ming mice (initial body weight, 18–22 g; 4 weeks old)	10	50, 100 mg/kg	4 weeks	Weight loading swimming time↑	T-SOD, CAT↑; MDA↓; Thymus index and spleen index↑; Spleen lymphocyte transformation ability↓; Urea nitrogen↓; Hepatic glycogen↑	(80)
LBP	*Lycium barbarum*	*In vivo*	Forty male Kun-ming mice (18–22 g)	10	10, 20 mg/kg/d	ay 4 weeks	Swimming endurance↑	Skeletal muscle damage↓, MDA↓; SOD, GSH-Px activities↑; Mitochondrial membrane potential, Ca^2+^↑	(146)
LBP, LBPT	*Lycium barbarum*	*In vivo*	Sixty-four male SD rats (4 weeks old; body weight 180–210 g)	8	LBP: 120, 360 mg/kg/day; LBPT: 600, 1,200, 1,800 mg/kg/day	28 consecutive days	Exhaustive swimming time↑	ALT, CK and LDH↓; AST, BUN, BLA↓; Antioxidant capacity↑; Serum glucose, ATP, glycogen content↑; Density of mitochondria↑	(82)
LBP1-SeNPs	*Lycium barbarum*	*In vivo*	160 male ICR mice (20 ± 2 g)	20	0.5, 2, 4 mg Se/kg/day	30 days	Exhaustion swimming time↑	Liver and muscle glycogen↑; BUN, BLA↓; SOD↑; MDA↓; Antioxidant enzymes levels↑	(147)
PYB-1, PYB-2	*Chinese yam bulbils*	*In vivo*	Mice (20–22 g)	18	50, 100, 200 mg/kg	28 days	Swimming time↑	Hepatic glycogen content, antioxidant enzyme (SOD, glutathione peroxidase (GSH-Px)) activity↑; BUN, lactic acid, malondialdehyde levels↓	(156)
CYP	*Chinese yam (Dioscorea opposita Thunb.)*	*In vivo*	Swiss mice, typically weighing 18–22 g for females	10	once daily, 100 mg/kg	14 days	Exhausting swimming time↑	ATP in musculus gastrocnemius↑; IL-lβ, MDA, BUN, LDH↓; SOD activity↑	(179)
ZOPA, ZOPA-1	*Zingiber officinale*	*In vivo*	SPF Kunming male mice (weighing 20.0 ± 2.0 g, derived from Swiss mice)	20	400, 800 mg/kg	21 days	Exhaustion swimming times↑	LG↑; LDH, BUN↓; Na^+^K^+^-ATPase and Mg^2+^-ATPase content↑; IL-1β and IL-6↓; MDA↓	(64)
PPPm-1	*Paris polyphylla*	*In vivo*	Healthy Kunming mice (18–22 g, *n* = 48, 24 males and 24 females)	12	100, 200, 400 mg/kg	21 days	Weight-bearing swimming time↑	Blood lactate, serum urea nitrogen↓; Hepatic glycogen, muscle glycogen↑; Endurance, glycogen reserve↑; Glycogen consumption, lactate, serum urea nitrogen accumulation↓; Ca^2+^ influx↑	(157)
EPDO-60	*Dendrobium officinale*	*In vivo*	Male Institute of Cancer Research mice (18–22 g, 6 weeks)	10	100, 150, 200 mg/kg, P60–H	4 weeks	Forced swimming time↑	BLA, BUN↓; SOD↑; Proportions of Bacteroidetes and Firmicutes and abundance of Lactobacillus and Bifidobacterium in gut microflora↑	(66)
BCP-2	*Bupleurum chinense DC*	*In vivo*	Healthy 2-month-old male ICR mice (20 ± 2 g)	10	50, 100, and 200 mg/kg	30 days	Forced swimming time↑	Glycogen reserves, antioxidant system↑; BUN, lactic acid, lactate dehydrogenase, creatinine kinase expression↓	(67)
PAP	*Apple pomace*	*In vivo*	Seven-week-old Kunming male mice (18–20 g)	6	50, 100, and 200 mg/kg	4 consecutive weeks	Exhaustive swimming time↑	Glycogen content↑; activity and gene expression of glycogen synthase↑; Hepatic and skeletal muscle glycogen↑; Blood lactic, BUN↓	(158)
APs	*A. membranaceus*	*In vivo*	Six-week-old C57BL/6 male mice (*n* = 40) weighting 20 ± 2 g	8	200, 400, and 800 mg/kg dose	5 weeks	Total distance of movement↑, central residence time↑, immobility time↓	SCFAs↑, anti-inflammatory bacteria↑	(73)
MCP	*Lepidium meyenii (Walp.)*	*In vivo*	male Kunming mice	10	150, 300, 600 mg/kg/day	30 days	Exhaustive swimming time↑	LG↑; BUN↓	(152)
MPs-1, MPs-2	*Lepidium meyenii Walp. (maca)*	*In vivo*	four-week old male Kunming mice	10	20, 100 mg/kg/day	30 days	Exhaustive time in forced swimming↑	BLA, BUN, LDH↓; LG↑	(154)
MP	*Lepidium meyenii Walp*	*In vivo*	ICR mice, including 40 males and 40 females, weighing 26.20 ± 1.70 g at 6 week of age	10	25, 50, and 100 mg/kg bw/day	30 consecutive days	Swimming durations↑, average swimming speeds↑	Glutathione peroxidase, creatine kinase activities↑; Lactate dehydrogenase activity↓; BUN, lactic acid, and malondialdehyde↓	(153)
ME	*Maca (Lepidium meyenii Walp.)*	*In vivo*	ICR mice (18–22 g)	10	10 ml/kg bw	4 weeks	Leg grip-strength↑, exercise endurance in the rota-rod test↑, mouse muscle structures↑	BLA, BUN, ROS↓; NAD+/NADH↑; Cell viability of C2C12 cells↑	(193)
AP	*Abelmoschus esculentus*	*In vivo*	Kunming male mice	40	0.8, 1.6, and 3.2 g/kg	2 weeks	Swimming time↑	Liver glycogen, serum lactic acid, and serum urea↑; Testicles and epididymis↑	(194)
AEP-1, AEP-2	*Okra (Abelmoschus esculentus (L.) Moench)*	*In vivo*	Male Kunming mice (5-week old, body weight 20 ± 2 g)	30	50, 100, and 200 mg/kg	4 weeks	Swimming time↑	Serum urea nitrogen (SUN), BLA ↓; hepatic glycogen (HG), muscle glycogen (MG) ↑; Creatine kinase (CK), lactate dehydrogenase (LDH) ↓; Succinate dehydrogenase (SDH), adenosine triphosphate (ATP), adenosine triphosphatase (ATPase)↑	(66)
PCS	*Corn silk*	*In vivo*	Healthy Kunming mice (aged 4 weeks, half male and half female, weight 20 ± 2 g)	20	50, 100, 200, and 400 mg/kg	2 weeks	Duration of the swimming time to exhaustion↑	BUN, LA↓, LDH, HG activities↑	(159)
U-SCPSeNP 0, U-SCPSeNP 20, U-SCPSeNP 40, U-SCPSeNP 60	*Sweet corn cob*	*In vivo*	Male five-week-old ICR mice (*n* = 64) weighing 20 ± 2 g	8	200 mg/kg	28 days	The production capacity of Na^+^-K^+^-ATP, Mg^2+^-ATP, Ca^2+^-ATP↑	SOD, MDA↓; CAT, GSH-Px↑; Diversity and abundance of gut microbiota↑; Relative abundance of Firmicutes↓, relative abundance of Bacteroidota↑	(195)
MSP	*Millettiae speciosae Champ. Leguminosae*	*In vivo*	Male Kunming mice, weighted 15–17 g	9	200, 400, and 800 mg/kg	20 days	Swimming time to exhaustion↑	Glucose (Glu), muscle glycogen↑; BUN, lactic acid (Lac) ↓; Creatine phosphokinase (CK), lactic dehydrogenase (LDH), malondialdehyde (MDA) ↑; Superoxide dismutase (SOD), glutathione (GSH)↓	(177)
RRPP	*Radix Rehmanniae Preparata*	*In vivo*	Male BALB/c mice (8 weeks old, 17–20 g)	20	50, 100, and 200 mg/kg	4 weeks	Exhausting swimming time↑	BLA, SUN↓; Hepatic glycogen↑	(196)
MOA	Roots of *Morinda officinalis*	*In vivo*	Male Sprague Dawley (SD) mice with a body weight range of 18–22 g	10	0, 50, 100, and 200 mg/kg	4 weeks	Weight-loaded swimming time↑	Serum urea nitrogen, BLA↓; Hepatic glycogen↑	(197)
GP	*Garlic*	*In vivo*	SPF adult male ICR mice (6–8 weeks; 16–18 g body weight)	20	1.25, 2.5 g/kg-BW	7 weeks	Duration of exhaustive swimming↑	Blood biochemical markers (BUN and BLA), liver and muscle glycogen↑; Antioxidant enzyme activity (SOD, GSH-Px, and CAT), ATPase activity↑; Potentially beneficial bacteria↑, harmful bacteria↓	(74)
PKP	*Polygonatum kingianum Collett & Hemsl*	*In vivo*	Male non-specific pathogen KM mice (20 ± 2 g, SPF), aged 5 weeks	12	100, 150, and 200 mg/kg/day	4 weeks	Exhaustive swimming time↑	Antioxidant bacteria (e.g., g_norank_f_Muribaculaceae), short-chain fatty acids (SCFAs) ↑; Abundance of harmful bacteria (e.g., g_Escherichia-Shigella and g_Helicobacter) ↓	(83)
PEP	*Phragmites rhizome*	*In vivo*	Male ICR mice (6 weeks old) and Sprague-Dawley rats (8 weeks old)	6	1 g/kg, p.o.	10 days	Swimming endurance capacity↑, body weight↑	GSH, SOD, CAT, GSH-Px↑; CK↓; Hyperactivation of the hypothalamus–pituitary–adrenal axis↓, oxidative damages induced by WIR stress↓	(64)
GPP1-a	*Gynostemma pentaphyllum*	*In vivo*	Male 2-month-old Kunming mice	10	50, 100, and 150 mg/kg	1 week	Exercise time to exhaustion↑	Glycogen level, some of antioxidant enzyme activities↑; MDA↓	(70)
ASP	*Angelica sinensis (AS)*	*In vivo*	W1118 (#5905) fly strains	20	0, 1,3 mg/mL	15 days	Lifespan↑, reproduction↑, climbing ability↑, resistance to starvation and oxidative stress↑	Insulin signaling (IIS), TOR signaling↓, antioxidant ability↑; Intestinal stem cells (ISCs) hyperproliferation and oxidative damage↓	(198)
CPP	*Cyclocarya paliurus (C. paliurus)*	*In vitro*	RAW264.7 cell line	1 × 105 cells/ml	3.125,6.25, 12.5, 25, 50, 100, 200, and 400 μg/ml	24 h	Cell viability↑, antioxidant activity↑	MDA↓; Activity of SOD, T-AOC, CAT↑	(199)
MHa	*Mentha haplocalyx*	*In vivo*	Male C57BL/6 mice (20 ± 2 g)	10	100, 200, and 400 mg/kg	30 days	Exhaustive swimming time↑	Blood lactate, urea nitrogen levels↓; Liver glycogen, muscle glycogen, ATP levels↑; Activities of Ca^2+^-Mg^2+^-ATPase and Na^+^-K^+^-ATPase, antioxidant defense↑	(160)
AALP-U	*Artemisia argyi leaves*	*In vivo*	healthy SPF-grade male ICR mice, 6–8 weeks old, 18–22 g	20	50, 100, and 200 mg/kg/day	30 days	Swimming time↑, exercise endurance↑	Liver and muscle glycogen↑; SOD, GSH-Px↑; ROS↓	(200)
LJP	*Laminaria japonica*	*In vivo*	Adult male Kunming mice (Mus musculus, Km, with weight 20 ± 2 g)	12	75, 150, and 300 mg/kg	28 days	Swimming time to exhaustion↑	Liver and muscle glycogen content, levels of superoxide dismutase, glutathione peroxidase, catalase in the serum, liver, and muscle↑; Malondialdehyde (MDA) ↓; BLA, serum myeloperoxidase (MPO) ↓	(201)
PSP	*Spirulina platensis*	*In vivo*	Adult male Sprague-Dawley rats of grade SPF, weighing 221.98 ± 22.67 g	10	50, 100, and 200 mg/kg	6 consecutive days	The time to exhaustion during the treadmill exercise↑	Hb levels↑; LA, BUN, and CK levels in the blood↓; 5-HT concentrations, TPH2 expression↓; 5-HT1B expression in the caudate putamen↑	(173)
SCPs	*Sweet cassava*	*In vivo*	Male Sprague–Dawley (SD) rats (5 weeks old and weighting 180–200 g)	10	500 mg/kg/day (in two 250 mg/kg doses; one after the morning exercise and the other in the evening at ~17:00–18:00)	5 days	Running time to exhaustion↑	Glycogen content in the soleus and gastrocnemius muscles↑; Blood glucose and free fatty acid (FFA) ↑	(202)
HRWP, LBWP, LRWP, NTWP	The fruits of *Hippophae rhamnoides, Lycium barbarum, Lycium ruthenicum, Nitraria tangutorum*	*In vivo*	Male BALB/c male mice (8 weeks old)	6	50, 100, and 200 mg/kg	15 days	Immobility in the FST↓	Glc, SOD, GSH-Px↑; CK, LDH, BUN, TG, MDA↓; Spleen indices↑(LBWP and NTWP)	(181)

## 5 Mechanisms of natural medicinal plant polysaccharides in enhancing aerobic exercise capacity

Natural medicinal plant polysaccharides enhanced aerobic exercise capacity through various mechanisms. The major mechanisms include microbiota modulation, energy metabolism regulation, antioxidation, anti-inflammation and immunity regulation.

### 5.1 Modulation of gut microbiota

#### 5.1.1 Modulation of gut microbiota composition

The composition and function of intestinal microbiota are important in energy metabolism homeostasis, and polysaccharides from natural medicinal plants can improve aerobic exercise ability by regulating the composition of gut microbiota. Many studies have shown that the structure, abundance, diversity in the intestine mediate the effect of polysaccharides from natural medicinal plants on aerobic exercise ability. Polysaccharides such as ZOPA and ZOPA-1 have been shown to modulate the intestinal flora of mice, increasing diversity, altering abundance, regulating short-chain fatty acid concentrations, and enhancing antioxidant capacity through the gut-muscle axis, extending the time of exhaustive swimming in mice (64). Moreover, *Ginseng* polysaccharides could regulate the gut microbiota composition and promote M2 macrophage polarization by modulating TLR4/MYD88 signaling (90).

Changes in the beneficial and harmful bacteria in the intestinal flora are also important factors for the influence of NMPPs on aerobic exercise ability through the intestinal muscle axis. Polysaccharides from *Dendrobium officinale* (EPDO-60) modulates gut microbiota community structure by increasing proportions of Bacteroidetes and Firmicutes and abundance of *Lactobacillus* and *Bifidobacterium* in gut microbiota, thereby enhancing redox homeostasis and accelerating fatigue-related metabolite clearance (65). HRP promotes beneficial bacteria like Clostridia_UCG-014, Lachnospiraceae and suppressed pathogens such as Atopostipes, Desulfobacterot, which is crucial in HRP-mediated immunity regulation via TRAF6/NF-κB signaling, up-regulated the expression of occludin, claudin-1, and zona occludens-1 (ZO-1) (91). The GRP supplement modulated the Firmicutes/Mycobacteria ratio and Blautia spp., significantly reducing oxidative stress and inflammation in the liver of mice fed a high-fat diet (106). PKPs can reduce the number of harmful bacteria (g_*Escherichia-Shigella* and g_*Helicobacter*), while increasing the production of antioxidant bacteria (g_norank_f_Muribaculaceae) and short-chain fatty acids (SCFA), and significantly enhance exhaustive swimming time (83).

#### 5.1.2 Metabolic regulation of gut microbiota

In addition to directly affecting the structure of intestinal flora, polysaccharides from natural medicinal plants can also improve aerobic exercise ability through the metabolites of intestinal flora. SCFAs are significant metabolites produced by gut microbiota during the fermentation of NMPP. A study showed that APs induced SCFAs generation and consequently reversing Nrf2/NF-κB signaling dysregulation in the brain-gut axis, which may be a key mechanism for ameliorating chronic fatigue syndrome-related metabolic disturbances (73). SCFAs also enhanced by ACP to modulate the microbe-gut-brain axis, increasing the lactic acid levels in colon contents, the abundance of Bacteroides while reducing Firmicutes and other harmful bacteria (108). Furthermore, multiple polysaccharides (e.g., FAPs, PKPs) enhanced SCFA production, linking microbial SCFA production to muscle energy optimization and improving gut barrier integrity and energy homeostasis (83, 162). MPS-NPs maintained Firmicutes/Bacteroidetes ratio and upregulated SCFA biosynthesis, such as butyrate production, which plays a critical role in maintaining gut health and energy homeostasis, supporting gut barrier integrity and muscle ATP production (79). Butyrate also mediated antioxidant and anti-inflammatory effects in neurons, linking gut ecology to systemic immunity (73). These findings position the gut as a metabolic orchestrator of muscle resilience (163). The enhanced SCFAs by AVFP is correlated with the increased beneficial bacteria, reduced harmful species, modulated immune responses through NF-κB signaling pathway, and improved the host ability to resist oxidative stress (95). In addition, studies have found that DOP increases taurine and decreases 2-hydroxybutyric acid, while promoting the growth of probiotics such as Dubosiella, Bifidobacterium, and Akkermansia, which also have beneficial effects on improving aerobic exercise capacity (164).

### 5.2 Energy metabolism in mitochondria and skeletal muscles

Energy metabolism, particularly in mitochondria and skeletal muscles, is another key area where NMPPs exert their effects. In skeletal muscle, the activation of AMPK can initiate the oxidative metabolic program of mitochondria (165, 166). AMPK is a crucial molecular target for skeletal muscle fiber type transformation (167), and can also perceive cellular energy status through direct interactions with ATP, ADP, and AMP (168). Many studies showed that natural medicinal palnt polysaccharides can ameliorate exercise-induced fatigue by rectifying mitochondrial dysfunction, restoring energy metabolism homeostasis, and counteracting oxidative stress. For example, APs-1 activated AMPK signaling to enhance mitochondrial biogenesis and glucose uptake, alleviated muscle fiber contraction (144). Additionally, APs can enhance autophagy and suppressing inflammation and oxidative stress in myocardial tissues through AMPK signaling pathway, thereby protecting against overexercise-induced injury (169). More profoundly, polysaccharides like BCP-2 have been shown to alleviate physical fatigue by regulating the AMPK and Nrf2 signaling pathways in skeletal muscles, thereby enhancing mitochondrial biogenesis and antioxidant defenses (67). LBP and LBPT enhanced biochemical markers (e.g., lactic acid, creatine kinase) and antioxidant capacity in rat serum and muscles, and reversed exercise-induced decreases in serum glucose, ATP, and glycogen, while regulating energy metabolism through the AMPK/PGC-1α pathway (82). LBP also modulated glucose and lipid metabolism, improved skeletal muscle atrophy via AMPK/PINK1/Parkin-mediated mitophagy, and repaired mitochondrial structure and function (170). Furthermore, PKPs promoted energy metabolism by upregulating the expression of AMPK/PGC-1α/TFAM signaling pathway proteins (83). GP increased antioxidant enzyme activity (SOD, GSH-Px, and CAT) and restored ATPase activity by activating the AMPK/PGC-1α pathway (74). APs accelerated sheep skeletal muscle stem cell (SMSC) differentiation by upregulating miR-133a, a microRNA that enhances MAPK/ERK activity and myoblast fusion (155). PCP stimulated neurogenesis through the CREB/BDNF/Akt pathway and augmented glucose uptake via AMPK-TXNIP modulation, synergistically supporting anti-fatigue effects (150). In addition, there are some pathways that are less well studied. PSP attenuated age-associated mitochondrial dysfunction by activating the PI3K/Akt/mTOR pathway, which promoted protein synthesis and reduced muscle atrophy (171). PKPs can promote energy metabolism by upregulating the expression of AMPK/PGC-1α/TFAM signaling proteins, while changing gut flora, and significantly improve various physiological indicators related to fatigue (83).

In addition to directly acting on energy metabolism, NMPPs also play a role in regulating energy metabolism by regulating hormones and enzymes. For example, *Polygonatum cyrtonema* polysaccharides (PCP) enhanced osteocalcin expression via the BMP-2/Smad1/Runx2 axis, activated p-CREB and p-HSL in skeletal muscle, mediated crosstalk between skeletal and muscular systems to regulate energy metabolism, improving lipid mobilization and ATP generation (109, 149). Additionally, Gavage with *panax ginseng* polysaccharides (PGP) for 10 days daily could inhibit the formation of MDA in the brain of chronic hypoxia model mice, increase the levels of ATP, ADP, TAP and AEC in hepatocytes, increase CK activities, the ratio of ATP/ADP and ATP/AMP in skeletal muscle, and protect mitochondria by inhibiting mitochondrial swelling and improving energy metabolism (172). *Okra* polysaccharide enhanced succinate dehydrogenase (SDH) and ATPase activity, accelerating ATP regeneration and reducing CK leakage (66). An acid polysaccharide from *Mentha haplocalyx* (MHa) promoted mitochondrial biosynthesis by activating AMPK, increased liver glycogen and muscle glycogen storage, and increased the activity of Ca^2+^-Mg^2+^-ATPase and Na^+^-K^+^-ATPase, significantly increasing the swimming time of mice at exhaustion (160). In addition, polysaccharides from natural medicinal plants can improve motor ability by regulating the central nervous system. *Polygonatum sibiricum* polysaccharide (PSP) increased hemoglobin levels, decreased LA, BUN and CK levels in the blood; inhibited the increase of 5-HT concentration and tryptophan hydroxylase (TPH2) expression induced by exercise, and prolonged the fatigue time during treadmill exercise (173). *Acanthopanax* polysaccharide (ACP) can reduce the activation level of ROS-NLRP3 in substantia nigra striatum and improve the motor ability of Parkinson's disease mice (174).

Plant polysaccharides can improve aerobic exercise ability by increasing energy metabolism substrates such as muscle glycogen and liver glycogen. Selenium-enriched *Lycium barbarum* polysaccharide (LBP1-SeNPs) extended swimming endurance via enhancing muscle glycogen reserves and GSH-Px activity (147). *Maca* polysaccharides could increase liver glycogen (LG) content in mice, had the significant anti-fatigue activity (152). *Apple pomace* polysaccharide (PAP) upregulated hepatic glycogen synthase activity, elevating liver and muscle glycogen stores while inhibiting BUN and LDH accumulation, which contributes to their anti-fatigue activity (158). Furthermore, *Ziziphus* polysaccharide (PPPm-1) reduced glycogen depletion during exhaustive exercise, concurrently suppressing lactate accumulation and promoting Ca^2^^+^-mediated muscle contraction efficiency (157).

### 5.3 Antioxidation

Polysaccharides can also effectively improve the antioxidant capacity and consequently improve the aerobic exercise capacity. PKPs can improve intestinal flora and energy metabolism, and enhance exercise ability by activating NRF2/HO-1 signaling pathway to reduce oxidative stress (83). The Keap1-Nrf2/ARE signaling pathway is often implicated in these antioxidant effects, as seen with HWE-JGLR, which enhances antioxidant function in broilers (175). In exhaustive swimming experiments with mice, both crude ZOPA and purified ZOPA-1 exhibited significant anti-fatigue effects, which may improve antioxidant capacity through the activation of the Keap1-Nrf2/ARE and AMPK/PGC-1α signaling pathways (64). Moreover, LBP protected retinal cells against light-induced damage by upregulating Nrf2 and thioredoxin reductase 1 (TrxR1), thereby neutralizing oxygen free radicals and reducing mitochondrial oxidative stress (176). The improvement of antioxidant capacity and the alleviation of oxidative stress by LBP may be achieved through Nrf2/HO-1 pathway (82). LBP1-SeNPs have been found to relieve fatigue by increasing glycogen reserves and enhancing antioxidant enzyme levels (147). LBP-4a (146) and total polysaccharides (MSP) from *Millettiae speciosae Champ. Leguminosae* (177) can reduce lipid peroxidation and increases antioxidant enzymes in skeletal muscle, improving calcium homeostasis and mitochondrial function.

The increase of antioxidant enzyme activity is also an important factor for the improvement of aerobic exercise ability of NMPPs. *Lycium barbarum* polysaccharide (LBP-4a) can enhance the anti-fatigue ability of subhealthy mice by reducing the lipid peroxidation level in skeletal muscle tissue, increasing the activity of SOD and GSH-Px, and improving the imbalance of intracellular calcium homeostasis (146). In a study on the mechanism of ginsenoside acid polysaccharide WGPA in the prevention of chronic fatigue syndrome (CFS), it was found that oral administration of WGPA for 15 days in mice could increase serum SOD and GSH-Px, improve mitochondrial structural degeneration, and prolong forced swimming time (81). RGP treatment activated the Nrf2/Keap1 pathway and significantly increased the activity of antioxidant enzymes (178). Similarly, APs enhances total antioxidant capacity, SOD, and GSH-Px activities in heart, kidney, and liver while lowering MDA levels, thereby reducing oxidative stress (76). Other compounds, such as CYP, ameliorated cisplatin-induced muscle atrophy by restoring ATP content, reducing oxidative markers (MDA, LDH), and elevating antioxidant enzymes (SOD) (179). Polysaccharides from *S. cassiae* have demonstrated strong anti-fatigue activity by ameliorating the levels of antioxidant enzymes such as SOD and GSH-Px while reducing markers of oxidative stress like malondialdehyde (MDA) (94). PCP has been shown to reduce oxidative stress markers and increase antioxidant enzyme activities, thereby exerting anti-fatigue properties (149). *Ziyang green tea selenium*-polysaccharide (Se-TP) (180) can alleviate exercise fatigue and prolong the time of exhaustion by increasing the content of GSH-PX, SOD and CAT, reducing the level of MDA, and increasing the content of muscle glycogen. PSP can effectively reduce the increase of Ca^2+^ concentration by regulating calcium-related proteins in muscle cells, reduce oxidative stress and mitochondrial dysfunction, increase muscle mass and improve grip strength and hanging time in old mice (148).

Notably, molecular weight is a key factor affecting their free radical scavenging activity and the effectiveness of enhancing athletic performance. For example, both WSGP-S3 (molecular weight 2.03 × 10^4^) and WSGP-G3 (molecular weight 4.86 × 10^4^) are acidic heteropolysaccharides extracted from steamed ginseng using ultrafiltration methods. However, only WSGP-S3 can extend the swimming endurance time of fatigued mice by increasing the activity of superoxide dismutase, catalase, and glutathione peroxidase, while reducing malondialdehyde levels (68). PYB-1 (molecular weight 145 kDa) and PYB-2 (molecular weight 11 kDa) are both polysaccharides from yam bulbils, but PYB-1 exhibits stronger free radical scavenging activity and can extend the intermittent swimming time of mice more effectively (156). A study compared the effects of *Hippophae rhamnoides* polysaccharides (HRWP), *Lycium barbarum* polysaccharides (LBWP), *Lycium ruthenicum* polysaccharides (LRWP) and Nitraria tangutorum polysaccharides (NTWP), and found that they can all inhibit glucose, SOD and GSH-Px in the liver and heart of mice induced by FST, showing anti-motor fatigue activity, and LBWP and NTWP are far better than HRWP and LRWP at the same dose (181).

### 5.4 Anti-inflammation

The polysaccharides of natural medicinal plants play a role in improving aerobic exercise ability by regulating inflammation and maintaining the stability of cell function. For example, HRP may regulate the expression of TRAF6 /NF-κB signaling pathway by affecting the diversity of intestinal microbiota and inhibiting the levels of pro-inflammatory cytokines (TNF-α, IL-6, IL-1β), up-regulated the expression of occludin, claudin-1, and zona occludens-1 (ZO-1) (91). GNP suppresses the expression of COX-2 and iNOS by blocking the MAPK/NF-κB signaling pathway (182). LBP combined with aerobic exercise can reduce the inflammatory factor related indicators of liver LPS/TLR4/NF-κB signaling pathway and improve liver inflammation in NAFLD (183).

### 5.5 Immunomodulation

The polysaccharides of natural medicinal plants can have a positive effect on aerobic exercise ability by improving immune function and enhancing fatigue recovery (184, 185). In vivo experiments have shown that DOP can enhance the production of sIgA and alleviate cyclophosphamide-induced immunosuppression by increasing immune organ indices, promoting immunoglobulin secretion, and boosting the number of immune cells (186). *Polygonatum* polysaccharide can enhance the immune regulatory activity, significantly improve the spleen index and thymus index, increase the expression of IL-2, IFN-γ, IgA and IgM, and increase the CD4+/CD8+ ratio, with significant immune regulatory effect (187). Polysaccharide AVFP regulates NF-κB-mediated immune responses by modulating the expression of Bcl3, Lbp, and Cebpd, suggesting its broad-spectrum immunoregulatory activity and ability to enhance the body's resistance to oxidative stress (95). Dietary APs has been shown to enhance immune functions in mice, increasing white blood cell and lymphocyte counts, upregulated genes associated with antioxidant defense and leukocyte proliferation (76). Moreover, APs improved growth performance, enhanced the antioxidant capacity and immune regulation of *Plectropomus leopardus* by regulating the expression of genes related to antioxidant enzymes and immune response via dose-dependent modulation (101). *In vitro* experiments have shown that *Hemerocallis citrina Borani* polysaccharide (HCBP1-1) can significantly promote the secretion of NO, TNF-α, IL-1β, and IL-6 in RAW264.7 cells, as well as the expression of NF-κB p65, demonstrating excellent immune-enhancing activity (188). Barbary wolfberry polysaccharides can increase the phagocytic ability and NO release in RAW264.7 cells by 23 and 76%, respectively, showing the strongest immune-enhancing activity (189).

**Figure 2:**
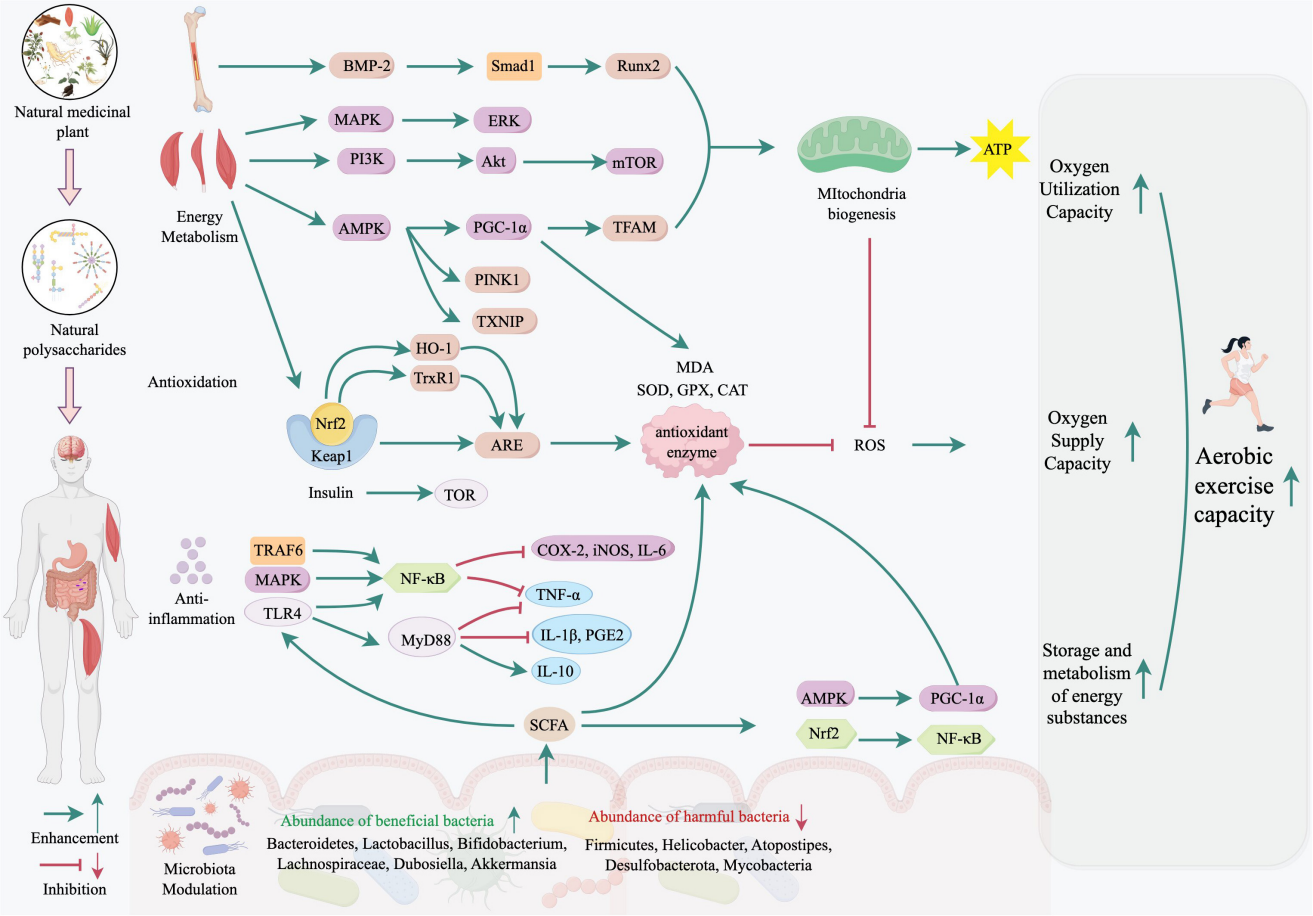
The mechanisms of NMPPs in enhancing aerobic exercise capacity NMPPs can enhance aerobic exercise capacity through the mechanisms of gut microbiota, energy metabolism in skeletal muscle mitochondria, antioxidation, anti-inflammation and immunomodulation.

In summary, NMPPs enhance aerobic exercise capacity through a multifaceted approach involving microbiota modulation, energy metabolism, metabolic regulation, antioxidation, inflammation and immunity, and organ crosstalk. These mechanisms collectively contribute to improved exercise performance, reduced fatigue, and enhanced recovery, making polysaccharides a promising area of research for athletes and individuals seeking to improve their physical endurance. The mechanisms of NMPPs in enhancing aerobic exercise capacity is shown in Figure 2.

## 6 Summary and perspective

In conclusion, NMPPs have emerged as highly promising bioactive compounds with significant potential to enhance aerobic exercise capacity. A wealth of research evidence has been comprehensively reviewed, unraveling the intricate mechanisms through which NMPPs exert their beneficial effects. NMPPs modulate gut microbiota to optimize energy harvest and intestinal homeostasis, fine-tune energy metabolism to boost mitochondrial function and fuel utilization, bolster antioxidant defenses to mitigate exercise-induced oxidative damage, and regulate inflammatory and immune responses to promote recovery and reduce fatigue. These multifaceted actions collectively result in improved aerobic exercise performance, delayed onset of fatigue, and enhanced post-exercise recovery. In addition, the structural diversity of NMPPs imparts them with unique bioactive properties. These structural features dictate their interactions with biological systems, influencing bioavailability, metabolic processing, and the specific signaling pathways they activate or suppress. Thus, a deeper understanding of the structure—activity relationships of NMPPs is crucial for harnessing their full potential in sports nutrition and exercise physiology.

While the existing body of research has laid a solid foundation for our understanding of NMPPs in the context of aerobic exercise capacity enhancement, several limitations and challenges warrant attention. The heterogeneity across experimental models, including variations in animal species, exercise protocols, and outcome measures, complicates the direct comparison of results and the derivation of generalized conclusions. Many exercise programs that test aerobic capacity use forced swimming to exhaustion. Moreover, most of the indicators observed in animal models are the improvement of fatigue state, and there are few studies on direct promotion in physiological health state. Furthermore, most studies have been confined to preclinical models, with limited clinical trials in humans. This gap raises questions about the expansion from preclinical findings to human athletic performance and the potential species—specific differences in the metabolic processing and bioactivities of NMPPs. Additionally, the long-term safety, optimal dosing regimens, and potential synergies or antagonisms between different NMPPs or with other nutrients remain to be elucidated.

Future research endeavors should focus on addressing these limitations through rigorous trials employing standardized protocols and well-defined NMPP preparations. Advanced omics technologies could be leveraged to dissect the molecular mechanisms underpinning the effects of NMPPs on aerobic exercise capacity at a systems level. Furthermore, exploring structure—activity relationships using sophisticated analytical techniques may pave the way for the rational design of NMPP—based supplements with enhanced efficacy and specificity. As the field progresses, NMPPs hold the promise of becoming a cornerstone in sports nutrition, offering athletes and physically active individuals a natural, safe, and effective strategy to optimize their aerobic exercise capabilities and overall athletic performance.
